# Uncoupling FRUITFULL’s functions through modification of a protein motif identified by co-ortholog analysis

**DOI:** 10.1093/nar/gkae963

**Published:** 2024-10-30

**Authors:** Kai Thoris, Miguel Correa Marrero, Martijn Fiers, Xuelei Lai, Iris E Zahn, Xiaobing Jiang, Mark Mekken, Stefan Busscher, Stuart Jansma, Max Nanao, Dick de Ridder, Aalt D J van Dijk, Gerco C Angenent, Richard G H Immink, Chloe Zubieta, Marian Bemer

**Affiliations:** Laboratory of Molecular Biology, Wageningen University & Research, 6708 PB Wageningen, the Netherlands; Bioinformatics Group, Wageningen University & Research, 6708 PB Wageningen, the Netherlands; Business Unit Bioscience, Wageningen University & Research, 6708 PB Wageningen, the Netherlands; Laboratoire de Physiologie Cellulaire et Végétale, CNRS, CEA, Université Grenoble Alpes, INRAE, IRIG, CEA, 38000 Grenoble, Grenoble, France; Laboratory of Molecular Biology, Wageningen University & Research, 6708 PB Wageningen, the Netherlands; Laboratory of Molecular Biology, Wageningen University & Research, 6708 PB Wageningen, the Netherlands; Laboratory of Molecular Biology, Wageningen University & Research, 6708 PB Wageningen, the Netherlands; Laboratory of Molecular Biology, Wageningen University & Research, 6708 PB Wageningen, the Netherlands; Laboratory of Molecular Biology, Wageningen University & Research, 6708 PB Wageningen, the Netherlands; Structural Biology, European Synchrotron Radiation Facility, 71 ave. des Martyrs, 38000 Grenoble, France; Bioinformatics Group, Wageningen University & Research, 6708 PB Wageningen, the Netherlands; Bioinformatics Group, Wageningen University & Research, 6708 PB Wageningen, the Netherlands; Laboratory of Molecular Biology, Wageningen University & Research, 6708 PB Wageningen, the Netherlands; Business Unit Bioscience, Wageningen University & Research, 6708 PB Wageningen, the Netherlands; Laboratory of Molecular Biology, Wageningen University & Research, 6708 PB Wageningen, the Netherlands; Business Unit Bioscience, Wageningen University & Research, 6708 PB Wageningen, the Netherlands; Laboratoire de Physiologie Cellulaire et Végétale, CNRS, CEA, Université Grenoble Alpes, INRAE, IRIG, CEA, 38000 Grenoble, Grenoble, France; Business Unit Bioscience, Wageningen University & Research, 6708 PB Wageningen, the Netherlands

## Abstract

Many plant transcription factors (TFs) are multifunctional and regulate growth and development in more than one tissue. These TFs can generally associate with different protein partners depending on the tissue type, thereby regulating tissue-specific target gene sets. However, how interaction specificity is ensured is still largely unclear. Here, we examine protein–protein interaction specificity using subfunctionalized co-orthologs of the FRUITFULL (FUL) subfamily of MADS-domain TFs. In Arabidopsis, FUL is multifunctional, playing important roles in flowering and fruiting, whereas these functions have partially been divided in the tomato co-orthologs FUL1 and FUL2. By linking protein sequence and function, we discovered a key amino acid motif that determines interaction specificity of MADS-domain TFs, which in Arabidopsis FUL determines the interaction with AGAMOUS and SEPALLATA proteins, linked to the regulation of a subset of targets. This insight offers great opportunities to dissect the biological functions of multifunctional MADS TFs.

## Graphical Abstract

**Graphical Abstract:**
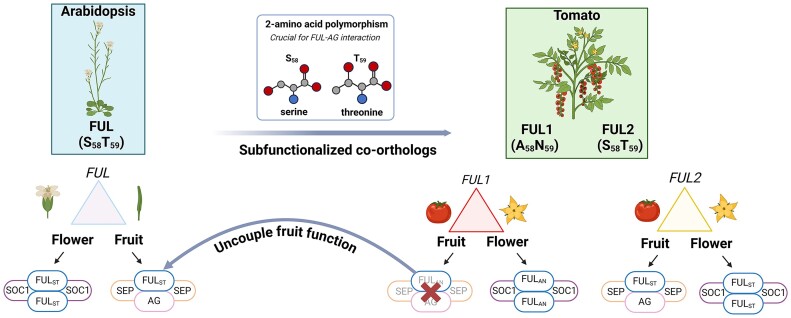

## Introduction

Many transcription-factor (TF) encoding genes are expressed in different tissues and influence multiple traits. These versatile TFs can generally associate with different protein partners, depending on the available tissue-dependent interactors. In plants, ubiquitous whole genome duplications (WGDs) (1) have resulted in a complex protein interactome, with closely related (paralogous) versatile TFs undergoing varying degrees of subfunctionalization, giving rise to unique and redundant or semi-redundant functions. Within large plant TF families, including the bHLH family, MADS-box and AP2/ERF families, this is especially common. For instance, the bHLH genes *INDEHISCENT* (*IND*), *SPATULA* (*SPT*) and *HECATE 1-3* (*HEC1-3*) have unique as well as overlapping functions in gynoecium patterning, shoot apical meristem specification and photomorphogenesis (2,3), the MADS-box genes *APETALA 1* (AP1), *CAULIFLOWER* (*CAL*) and *FRUITFULL* (*FUL*) act partially redundantly in shoot apical meristem, stem and fruit development (4–7), and the AP2/ERF family genes *APETALA 2* (*AP2*), *SCHLAFMÜTZE* (*SMZ*), *SCHNARCHZAPFEN* (*SNZ*) and *TARGET OF EAT 1-3* (*TOE1-3*) have redundant and separate functions in flowering time regulation, floral organogenesis and fruit development (8–10).

Gene multifunctionality reflects the efficient use of existing networks in different tissues, but the interconnectivity between processes prevents trait-specific adaptation, thereby resulting in evolutionary constraints (11,12). This interconnectivity is also a challenge in plant breeding, as mutations in versatile genes lead to undesirable pleiotropic phenotypes. Ideally, breeders have tools available to uncouple the functions of versatile genes and target-specific functions without disturbing others. Examining the *cis-*regulatory region to identify tissue-specific elements is a valuable approach to dissect gene functions that has started to yield results in recent years (13–16). Functional dissection at the protein level, however, has so far not been reported, although this is potentially a powerful strategy to target-specific functions, as TFs often interact with different proteins in different tissues (17–19). Linking protein motifs to tissue-specific functions is not straightforward though, as it requires detailed knowledge about the amino acids responsible for particular protein–protein interactions. We reasoned that investigating protein divergence of subfunctionalized paralogs can facilitate the identification of these amino acids, and thereby enable uncoupling of pleiotropic functions of orthologous multifunctional genes in other species. Here, we explored this approach and describe a successful method to dissect the functions of versatile TFs based on subfunctionalization in co-orthologs.

As a proof of principle, we focused on the versatile TF FUL. FUL belongs to the MIKC-type subfamily of MADS-domain TFs, of which the members are involved in numerous plant developmental pathways (reviewed in (18)). Proteins of this subfamily are characterized by the presence of four conserved domains: the MADS (M), intervening (I), keratin-like (K) and carboxyl-terminal (C) domains (20). To perform their functions, MADS-box TFs form dimers or tetramers with each other, mainly via their I- and K-domains, enabling them to bind with their M-domain to the DNA at so-called CArG-boxes (CC[A/T]_6_GG) (20). Despite the shared CArG-box-binding site, it seems that distinct MADS–domain complexes partially regulate different target gene sets (18,21–24). Recently, some studies have identified aspects that contribute to specificity, including minor differences in CArG-box sequence preference and DNA shape readout (18,22,23,25,26). Arabidopsis *FUL* is the ultimate example of a multifunctional gene, regulating silique- and cauline leaf development (5), flowering time (4), plant architecture (27) and life span (28). It belongs to the euFUL clade of the angiosperm-specific AP1/FUL gene lineage. Notably, two duplication events occurred early in core eudicot evolution and led to the emergence of three clades: AP1, euFULI and euFULII (29,30). Arabidopsis *FUL* belongs to the euFULI clade, while Arabidopsis *AGL79*, a gene that is hardly expressed, resides in the euFULII clade (30,31).

In several other eudicot families, duplication events led to the emergence of multiple copies of *FUL*, in which ancestral pleiotropy has been reduced through subfunctionalization (30). In the Solanaceae family, an early duplication in the euFULI clade resulted in two co-orthologs in all species, giving rise to a *FUL1* and *FUL2* lineage. A duplication event also occurred in the euFULII clade, which gave rise to two sub-clades, containing the *MBP10-* and *MBP20-*like genes (31). Together, the tomato genes have similar functions to those of the versatile *FUL* in Arabidopsis and play a role in flowering time, inflorescence architecture, fruit development and ripening (32,33). In particular FUL1 has subfunctionalized, exhibiting a reduced set of protein–protein interactions and loss of early fruit function (32,33).

Here, we linked the polymorphisms present in the tomato co-orthologs FUL1 and FUL2 to differences in their protein functions, and subsequently used these results to uncouple the different functions of their multifunctional Arabidopsis ortholog FUL. We show that a two-amino-acid polymorphism between FUL1 and FUL2 determines the fruit-specific physical interactions with AGAMOUS (AG) and SEPALLATA (SEP), while the interaction with the meristem-specific SUPPRESSOR OF OVEREXPRESSION OF CONSTANS (SOC1) is independent of this polymorphism. DAP-seq experiments reveal differences between the target gene sets of FUL-AG-SEP, FUL-SOC1 and FUL-FUL, linked to differential binding site preferences. In planta, modification of the two amino acids in FUL specifically leads to reduced functioning in the fruit. We additionally show that the two-amino acid polymorphism is part of a 12-amino acid motif that is more generally involved in the determination of MADS–domain interaction specificity. Given the high number of plant genomes sequenced in recent years (34) in combination with the ubiquitous lineage-specific WGDs, we expect that our approach can be generally used to uncouple functions of versatile TFs at the protein level.

## Materials and methods

### Electrophoretic mobility shift assay

Electrophoretic mobility shift assay (EMSAs) were performed according to Smaczniak et al. (17) with minor modifications. All coding sequences (FUL, SOC1, AG, SEP3, FUL-L-GFP, FUL-L-3xFLAG, FUL_AN_, FUL1, FUL2, TAG1, FUL_SD_, FUL1_ST_(C9), FUL1_AD_, FUL1_AT_, and FUL2_AN_(C8)) were cloned into a pSPUTK (Stratagene) protein expression vector. Proteins were produced *in vitro* with the TnT®SP6 High-Yield Wheat Germ Protein Expression System (Promega, L3260) according to the manufacturer's instructions. All used DNA probes were approximately 100 bp in length, amplified from genomic DNA and subsequently cloned into a pJET vector (Thermo Scientific, K1231). Following this, PCR amplification was conducted using DY-682 labeled primers targeting the vector backbone and the reactions were column purified (MACHEREY-NAGEL 740609.50S). For the supershift EMSA, 1 μl of Anti-FLAG Antibody (Miltenyi Biotec, 130-101-572) was added to the EMSA binding reaction 30 min after the start of incubation. All EMSA gels were visualized using a LiCor Odyssey imaging system at 700 nm.

### DAP-seq

The method was performed as previously described (24,35) with slight modifications. The experiment was conducted for the dimers/tetramers of interest, FUL-SOC1, FUL-AG-SEP3 and FUL-FUL alongside FUL-AG and FUL-SEP3 as control samples. Additionally, an input sample was included to correct for background signals. Proteins were produced *in vitro* with the TnT®SP6 High-Yield Wheat Germ Protein Expression System (Promega L3260) according to the manufacturer's instructions. All proteins were cloned into a pSPUTK (Stratagene) protein expression vector, with only a 3×FLAG tag attached to FUL. For single protein mixtures (e.g. FUL), 2 μg plasmid was used as input while for heterogeneous protein mixtures (FUL-SOC1, FUL-AG-SEP3, FUL-AG and FUL-SEP3), an equimolar ratio of plasmid was used, up to a total of 2 μg. All subsequent steps were performed at room temperature, and DNA LoBind® tubes (Eppendorf 0030108051) were used to maximize sample recovery. The *in vitro* produced proteins (48 μl) and DNA library (400 ng) were incubated in a total volume of 360 μl EMSA binding mix (17) for 2 h to ensure optimal protein/DNA binding conditions. The EMSA binding reaction was then added to 20 μl of washed anti-FLAG® magnetic beads (Sigma-Aldrich M8823), and the volume was increased to 1mL by adding lysis buffer (Miltenyi Biotec 130-091-125) with protease inhibitor (Roche 11697498001). This mixture was then incubated for 2 h on a tube revolver rotator (Thermo Scientific 88881001), followed by three washing steps with 400 μl 1× TBS. Next, the bound proteins were eluted from the beads by incubation with 400 μl 1× TBS buffer containing 150 ng/μl FLAG peptides (APExBIO A6001) for 45 min on a tube revolver after which the supernatant was collected on a magnetic stand. Subsequently, a second elution step was similarly performed to obtain a total volume of 800 μl. The collected supernatant was then incubated for 10 min at 95°C, immediately followed by column purification (MACHEREY-NAGEL 740609.50S) and elution with 50 μl Elution Buffer. The eluted DNA fragments were amplified for 20 cycles with Q5® High-Fidelity DNA Polymerase (NEB M0491) using Illumina TruSeq adaptors with unique barcodes and afterwards, they were purified with AMPure XP beads (Beckman A63880). The purified samples were loaded onto a 1% agarose gel and fragments ranging from 250 to 600 bp were cut out, purified from gel (MACHEREY-NAGEL 740609.50S), and subjected to another purification step using AMPure XP beads. The samples with different barcodes were pooled in equimolar ratios and sequenced with NovaSeq (Novogene) for 150 cycles using paired-end sequencing. Approximately 10–30 million reads were obtained for each sample and every experimental condition was done in triplicate.

#### Bioinformatic analyses

The reads were trimmed with Trim Galore (https://github.com/FelixKrueger/TrimGalore) and mapped to the Arabidopsis genome (TAIR10) with HISAT2 (36). Following this, the BAM files were used for MACS2 peak calling (37) with a significance threshold of *P*< 0.05 or *P*< 0.0001 and for differential peak analysis with MACS2 bdgdiff (37). The peaks were then annotated with ChiPseeker (38). To obtain motif files, we performed additional peak analysis with MEME (39) and GEM (21). The IGV genome browser (40) was used to visualize all peaks and sequencing data.

### Yeast two-hybrid

Yeast two-hybrid assays were performed exactly as described (41). Yeast clones containing MADS-domain coding sequences (CDSs) in either pDEST22 (AD-vector, Invitrogen) or pDEST32 (BD-vector, Invitrogen) were either obtained from the collection of De Folter and Immink, 2011, or cloned using the Gateway system and transformed into yeast strains PJ69-4A (pDEST22) and PJ69-4α (pDEST32). To swap amino acids between protein sequences, PCR fragments were combined using overhang-extension PCR. The oligos for PCR amplification and cloning are listed in Supplementary Table S4, and the sequence of the mutated proteins is listed in Supplementary Table S5. The interaction screen was performed on LWH dropout medium, supplemented with different concentrations of 3-amino-1,2,4-triazole (3-AT). Plates were incubated at RT and imaged after 3–6 days.

### Generation of Arabidopsis overexpression and complementation lines

Arabidopsis plants were grown in a growth chamber with 70% relative humidity on rockwool plugs (Grodan) at 20°C under LED light (150 μmol m-2 s -1). They were on a 16 h light/8 h dark day and night cycle and were watered twice a week with Hyponex fertilizer (1 g/L). All plants used in this study were in the Col-0 background, including the complementation lines, overexpression lines and the *ful-7* mutant (SALK_033647). All constructs for the complementation lines were generated with Gateway recombination cloning (Invitrogen); some were preassembled in Golden Gate or GreenGate vectors (42,43). The primers are listed in Supplementary Table S4. The native Arabidopsis FUL promoter (5.2kb) and terminator (1.2kb) were fused to the coding sequence of FUL (AT5G60910), FUL1 (Solyc06g069430), FUL2 (Solyc03g114830), MBP10 (Solyc02g065730) or MBP20 (Solyc02g089210). The complementation lines with switched amino acids (FUL_AN_, FUL1_ST_, FUL2_AN_) were obtained by introducing mutations with overlap extension PCR and/or Golden Gate cloning. Subsequently, the plasmids were transformed into *Agrobacterium* strain C58C1 and transformed in the *ful-7* mutant (SALK_033647) background.

### qRT-PCR analysis

Five stage 12–16 siliques (44) (positions 5–10) of the main inflorescence were harvested from each independent line (single plant). RNA was extracted with CTAB/LiCl and DNase-treated (TURBO™ DNase, Invitrogen, AM1907). cDNA synthesis was performed with SuperScript™ II Reverse Transcriptase (Invitrogen, 18064014). RT-qPCR was performed for *FUL*, *FUL1* and *FUL2* and the reference gene *UBC21* with iQ™ SYBR® Green (Bio-Rad, 1708880), using a standard two-step program of 40 cycles and an annealing temperature of 60°C. The primer efficiency was determined for all primer pairs and only those with equal efficiencies were used. All used primers can be found in Supplementary Table S4.

### I-domain motif identification

MIKC protein sequences from 1006 plant species were collected from different databases using hmmsearch v3.2.1 (45) to scan for sequences containing MADS- and K-domains using the pertinent Pfam models (PF00319.18 and PF01486.17, respectively) (46). After filtering out proteins with incomplete domains, a multiple sequence alignment was generated with Clustal Omega v1.2.4 (47). Sequences with uncommon length (<1.5× above or below the median SD) were filtered out, resulting in an alignment with 11966 MIKC-type protein sequences. Subsequences in between the detected M- and K-domains were considered to be the I regions. We used MEME v5.0.5 (48) to mine motifs in the collected I regions. We allowed for up to 75 different motifs, and we set a minimum motif width of four residues and a maximum width of 75. We used MEME with the zero or one motif occurrence per sequence model option (i.e. MEME assumes that each sequence may contain at most one occurrence of each motif). Subsequently, FIMO 5.0.5 (39,49) was used to search for individual occurrences of the discovered motifs in the I and C-terminal regions.

### Structural predictions

Structures of MADS complexes (homo- and heterodimers) were predicted using Colabfold in AlphaFold-multimer mode (50,51). Five models were generated for each MADS–domain TF complex and visually inspected. For all complexes, the top scoring model based on IPTM score was accepted, as it generated the correct DNA-binding domain based on the X-ray crystal structure of the SEP3 DNA-binding domain homodimer from Arabidopsis (PDB 7NB0; rcsb.org (52)). The AF2 models were then submitted to the Protein Interfaces Surfaces and Assemblies (PISA) server at the EBI for calculation of the buried surface area and the solvation free energy gain on formation of the interface, ΔG (53,54).

## Results

### Arabidopsis FUL performs its multiple functions by associating with distinct protein partners

**Figure 1.**
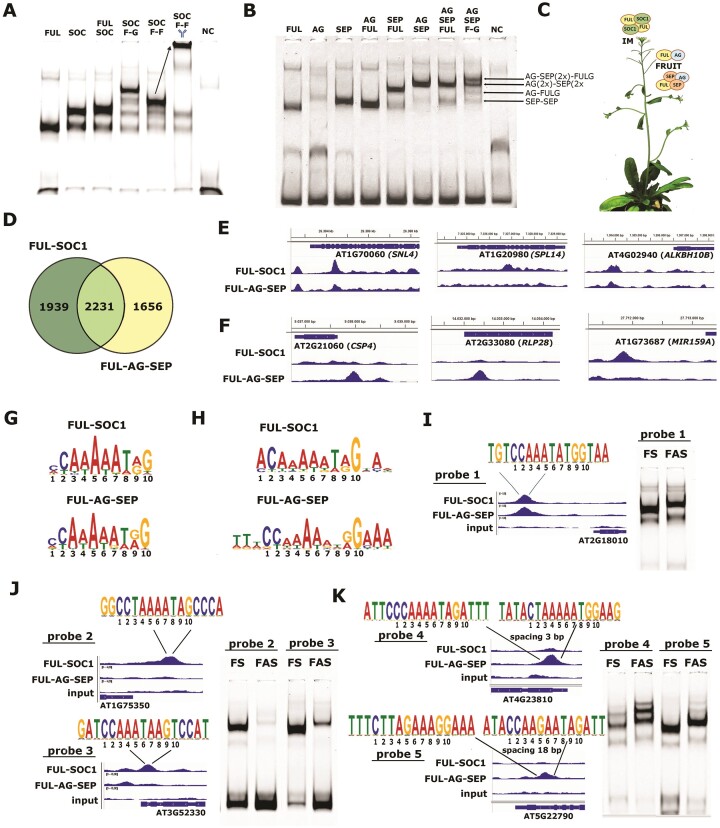
FUL performs its multiple functions by forming distinct complexes that regulate different target gene sets. (**A**) EMSA using the SAUR10 probe with a single canonical CArG-box (27) and different combinations of the FUL and SOC1 proteins. The 'Y' above the right lane with SOC F-F indicates the addition of FLAG antibody, the arrow indicates the shift of the complex that includes the antibody (tetramer of SOC1(2x) and FUL-FLAG (2x)). While FUL can form homodimers and SOC1 both homodimers and homotetramers, the clear shifts in the right lanes show that when FUL and SOC1 are combined, the FUL-SOC1 tetramer is most prominent. (**B**) EMSA similar to (**A**), but with FUL, SEP3 and AG proteins. The FUL homodimer migrates somewhat faster than the AG and SEP homodimers (lane 1–3). FUL forms a strong dimer with AG (lane 4), and predominantly a tetramer in combination with SEP3 (lane 5). AG-SEP can also form a tetramer, which migrates a bit slower than FUL-SEP (lane 6). When FUL, SEP and AG are combined (lane 7), the test with FUL-GFP (lane 8) indicates that the following complexes are predominantly formed: SEP homodimer, FUL-AG dimer, AG-SEP-AG-SEP tetramer and the FUL-SEP-AG-SEP tetramer. Abbreviations: SOC, SOC1; SEP, SEP3; F-G or FULG, FUL-linker-GFP; F-F, FUL-linker-FLAG; NC, negative control (probe with only TNT reaction mixture). (**C**) Drawing indicating that a FUL-SOC1 tetramer is the dominant complex in the IM, while complexes consisting of FUL, AG and SEP are dominant in the silique (fruit). (**D**) Venn diagram showing the unique and overlapping targets of the DAP-seq with FUL-SOC1 (three replicates combined) and FUL-AG-SEP (three replicates combined) for *P* < 0.05. (**E, F**) Genome browser snapshots of three genes that are significantly enriched (*P*< 0.0001) for FS (**E**) and three that are significantly enriched for FAS (P < 0.0001) (**F**). The *y*-axis indicates the normalized read counts based on the average of three replicates vs three input replicates. In most cases, the scale on the *y*-axis ranges between [0 and 4], only in the case of RLP28, the peak is higher, and the scale is [0–10]. (**G, H**) Sequence logos of the PWMs determined with MEME using all enriched loci for FUL-SOC1 (top) or FUL-AG-SEP (bottom) (**G**), or only the top 100 differentially enriched peaks (**H**). (**I-K**) EMSA experiments with FUL-SOC1 (FS) and FUL-AG-SEP (FAS) to test the binding to probes with different types of CArG-boxes (indicated above the peaks). (**I**) The SAUR10 probe with canonical CArG-box; (**J**) Probe fragments preferentially bound by FUL-SOC1 in DAP-seq; (**K**) Probe fragments preferentially bound by FUL-AG-SEP in DAP-seq.

The multifunctionality of Arabidopsis FUL is reflected in its protein–protein interaction pattern, as it can, among others, interact with the MADS-domain TFs SEPALLATA 1 (SEP1), SEP2, SEP3, SEP4, AGAMOUS (AG) and SUPPRESSOR OF OVEREXPRESSION OF CONSTANS 1 (SOC1) (17,55,56). FUL forms distinct complexes in the tissues where it executes important functions, associating with SOC1 in the inflorescence meristem (IM) and with AG and SEP1-3 in the fruit (18). To determine the preferred complex compositions of FUL in the IM and fruit, we performed EMSAs with a canonical CArG-box containing probe (27) and different combinations of FUL/SOC1 (Figure 1A) and of FUL/AG/SEP3 (Figure 1B). To distinguish between dimers and tetramers of similar size, we also included FUL-GFP (F-G) and FUL-FLAG (F-F) proteins and added anti-FLAG antibody to obtain a supershift for complexes that included FUL. This revealed that the FUL-SOC1 heterotetramer is most prominent when FUL and SOC1 are mixed while combining FUL, SEP3 and AG yields different complexes of which the FUL-AG dimer and FUL-SEP3-AG-SEP3 tetramer appear to be the most abundant FUL-containing complexes (Figure 1A–C; Supplementary Figure S1). Thus, in the IM, the FUL-SOC1 tetramer binds to target genes, while in the fruit, the FUL-AG dimer and FUL-SEP-AG-SEP tetramer probably bind most prominently to regulate their expression (Figure 1C). Hereafter, we will refer to these as the FUL–SOC1 (IM) and FUL–AG–SEP (fruit) complexes.

To investigate whether FUL-SOC1 and FUL-AG-SEP are binding to different target gene sets, we performed DAP-seq (24,35) with FUL-FLAG proteins either in combination with SOC1 or in combination with AG and SEP3. In total, 4170 significant binding events were identified for the DAP-seq with FUL-SOC1 and 3887 for FUL-AG-SEP at *P*< 0.05 (Supplementary Table S1), and 628 and 964 events at *P*< 0.0001 (Supplementary Table S2). To quickly assess the nature of the identified target loci, we performed a GO-term enrichment analysis, which revealed ‘carpel formation’ and ‘floral meristem determinacy’ among the most enriched biological processes (Supplementary Figure S2) and among the most enriched molecular functions ‘DNA-binding transcription factor activity’ (Supplementary Figure S3), reassuring us of the quality of the analysis. As expected for TF complexes, most binding events were located in the promoter regions of target genes (Supplementary Figure S4). Because both complexes are supposed to bind to CArG-boxes, we were not surprised that 2231 binding events overlapped between the FUL-SOC1 and FUL-AG-SEP datasets. However, a considerable number of binding events appeared only in the list of FUL-SOC1 or FUL-AG-SEP at cut-off *P*< 0.05 (Figure 1D). Varying the significance threshold resulted in shifts in the composition of the ‘unique’ and ‘overlapping’ lists, as a considerable number of the ‘unique’ peaks reflected different peak heights rather than a clear ‘binding’ versus ‘no-binding’ situation. By inspecting the list of targets at *P*< 0.0001 (Supplementary Table S2), we identified interesting targets where a clear binding event was detected for only one of the two complexes. For example, in the FUL-SOC1 data, significant peaks were detected for *SQUAMOSA PROMOTER BINDING PROTEIN-LIKE 14* (*SPL14*), a gene involved in plant architecture and flowering time (57); in *SIN3-like 4* (*SNL4*), which acts in histone deacetylase complex HDAC19 to repress flowering under short-day conditions (58); and in *ALKBH10B*, which encodes an RNA N6-methyladenosine demethylase that plays a role in the floral transition (59) (Figure 1E). This suggests that FUL-SOC1 may indeed bind certain flowering genes with a higher affinity than FUL-AG-SEP. However, this analytic approach yielded a number of putative differential binding events that were highly dependent on the selected significance threshold and sometimes reflected only minor differences in binding affinity, such as for example the peak at *ALKBH10B*, which was significant for both complexes at *P*< 0.05 but only for FUL-SOC1 at *P*< 0.0001 (Figure 1E). Therefore, we performed a differential read count analysis and sorted for the targets with the highest fold change (Supplementary Table S3). Strongly differentially bound loci for FUL-AG-SEP were for example the receptor-like protein *RLP28* and the cold shock domain protein *CSP4* (Figure 1F), with the latter being associated with silique growth (60). For FUL-SOC1, in particular a region upstream of *MIR159A*, which targets several MYB-domain TFs (61), was highly enriched (Figure 1F). Thus, although the sets of *in vitro* bound targets by FUL-SOC1 and FUL-AG-SEP are largely overlapping, distinct differences are also evident, and these can to some extent be linked to the putative biological functions of the two complexes.

Interestingly, we did not observe a clear difference between the sequence logos of the calculated position weight matrices (PWMs) for FUL-SOC1 and FUL-AG-SEP based on all targets (Figure 1G) but identified two clearly different PWMs when using the top 100 most differentially bound sites (Figure 1H). In the FUL-SOC1 PWM, there is a relaxed constraint for the fixed C at position 1 of the canonical motif (**C**C[A/T]_6_GG), which is remarkable, as this C is overall highly conserved in the binding motifs of different MADS–domain complexes (25). The PWM based on the specific FUL-AG-SEP targets on the other hand, displays more constraints than the canonical CArG-box motif, and A/T extensions at both the 5′ and 3′ end are added to the PWM (Figure 1H). To prove that FUL-SOC1 and FUL-AG-SEP bind to different sites, we performed EMSA experiments with five distinct probe fragments selected from the DAP-seq. Both FUL-SOC1 and FUL-AG-SEP bound to the canonical *SAUR10* CArG-box probe (27) with similar affinity (Figure 1I), but only FUL-SOC1 was binding with high affinity to the ‘relaxed’ CArG-box sites present in probe fragments 2 and 3 (Figure 1J). Probe fragments 4 and 5 were amplified from loci that were specifically bound by FUL-AG-SEP in the DAP-seq, and each contains two CArG-boxes, which are spaced 3 and 18 bp apart, respectively (Figure 1K). One of the CArG-boxes in probe 4 is very similar to the FUL-AG-SEP-specific PWM, and this probe was indeed bound stronger by FUL-AG-SEP. The CArG-boxes in probe 5 each contain a C/G in the conserved A/T core of the box (see Figure 1K), which is generally considered to completely prevent binding (25,27). However, the EMSA shows that both FUL-SOC1 and FUL-AG-SEP are able to bind with similar affinity to this probe, probably supported by the perfect A/T extensions at both ends of the CArG-boxes. Thus, although the contribution of flanking A/T nucleotides has been shown before (25,26), it appears that these are particularly important for the binding of FUL-AG-SEP, while FUL-SOC1 binds generally with high affinity to various CArG-boxes, with less dependence on the flanking nucleotides. Nevertheless, its binding to disturbed CArG-boxes (with C/G in the A/T core) may be enabled by A/T extensions on both sites as well, as exemplified by probe 5. The discrepancy between the DAP-seq and EMSA results for probe 5 may be explained by differences in DNA shape between the genomic-based DAP-seq fragments and the PCR-based EMSA fragments, because DNA shape read-out has been shown to contribute to binding affinity as well (22,23,26). In conclusion, Arabidopsis FUL associates with different MADS-domain proteins and these MADS–domain complexes differ in their ability to bind distinct CArG-box motifs, which likely contributes to a differential target gene regulation and allows the specific FUL-complexes to execute their functions in flowering time regulation and fruit development.

### The tomato FUL proteins differ in their interaction patterns and function

**Figure 2.**
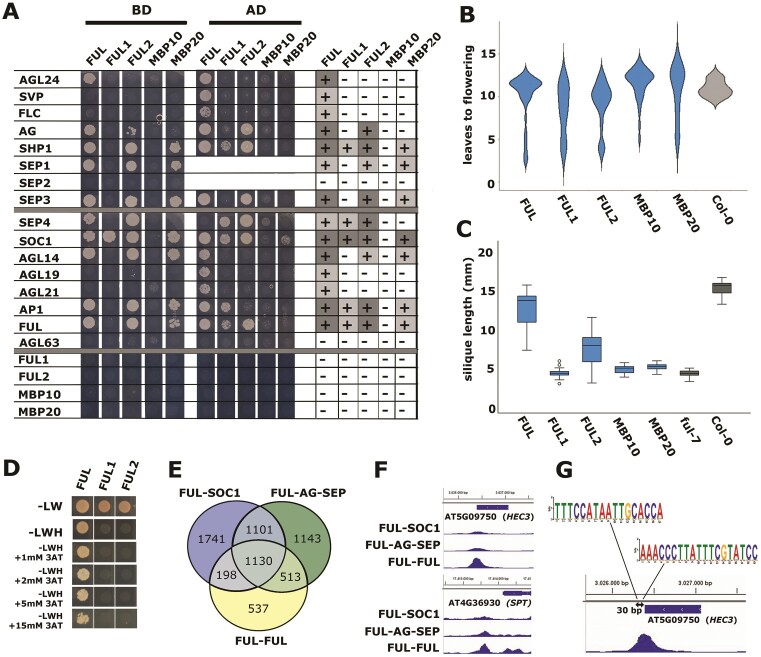
The FUL-like proteins have different protein–protein interaction patterns and differ in their ability to function in the Arabidopsis fruit. (**A**) Yeast two-hybrid experiment showing the physical interactions of the FUL-like proteins from tomato (FUL1, FUL2, MBP10, MBP20) and Arabidopsis (FUL) with a set of Arabidopsis MADS-domain proteins. The columns on the right summarize the data from both orientations (AD versus BD, BD versus AD). The screening was performed with 5mM 3-AT on -LWH plates. Because the fusion of the AD- or BD-domain to the N-terminus of the MADS-domain protein can affect interactions (e.g. (55)), we performed the screening in both orientations (indicated above panel A). SEP1-BD, and possibly also SEP2-BD, give autoactivation, and those were therefore only screened fused to the AD domain. In the case of SEP3-BD, a truncated version lacking the C-terminal end was used that does not display autoactivation. (56). (**B**) Violin plot showing the flowering time distribution of independent 35S:thFUL lines, measured as the number of leaves before flowering. For each construct, at least 30 transgenic lines were phenotyped. *x*-axis, FUL = 35S:FUL, FUL1 = 35S:FUL1, etc. (**C**) Box plots showing the silique length distribution of independent pFUL:thFUL:tFUL lines in the ful-7 mutant background. For each construct, at least 15 transgenic lines were phenotyped. *x*-axis: FUL = pFUL:FUL:tFUL, FUL1 = pFUL:FUL1:tFUL, etc. (**D**) Yeast two-hybrid to test the homodimerization of FUL, FUL1 and FUL2 at different concentrations of the competitor 3-AT. (**E**) Venn diagram showing the unique and overlapping significant targets (*P*< 0.05) of DAP-seq data sets from FUL-FUL, FUL-SOC1 and FUL-AG-SEP (each three replicates combined). (**F**) Genome browser snap shots of FUL-FUL, FUL-SOC1 or FUL-AG-SEP at the HEC3 and SPT loci. The *y*-axis indicates the normalized read counts based on the average of three replicates vs three input replicates. In the case of SPT, the *y*-axis ranges from [0–4], in the case of HEC3, this is [0–15]. (**G**) non-canonical CArG-boxes present under the HEC3 peak.

Tomato contains four *FUL*-like genes, which result from two Solanaceae-specific duplication events, one in the euFULI-clade and one in the euFULII-clade (31). The four tomato FULs display functional divergence, which is reflected in their protein–protein interaction profiles (32). While the multifunctional tomato FUL2 has a broad interaction pattern, in agreement with its different functions during flowering, fruit development and fruit ripening (32,33), the subfunctionalized FUL1 and MBP20 display more specific profiles and the non-functionalizing MBP10 hardly interacts at all (32). Interestingly, FUL2 can interact with the tomato ortholog of AG, named TOMATO AG 1 (TAG1), while its paralog FUL1 cannot (32). On the other hand, both can interact with the tomato co-orthologs of SOC1, TOMATO MADS 3 (TM3) and SISTER OF TM3 (STM3) (32,62), suggesting that FUL1 has subfunctionalized despite its high protein similarity with FUL2 (Supplementary Figure S5). We confirmed that tomato FUL1 has subfunctionalized compared to Arabidopsis FUL by testing the protein–protein interaction capacity of the tomato FUL-like proteins against a set of known Arabidopsis FUL interactors (Figure 2A). In agreement with the results of the tomato interaction studies, FUL1 could not interact with AG and had a more limited number of interactors than FUL2, which could interact with AG, SEP3 and seven other proteins. Similarly, MBP20 had overall less interaction partners than FUL2 and could not interact with AG. FUL1, FUL2 and MBP20 could all strongly interact with SOC1, indicating that the polymorphisms in their amino acid sequences do not affect general interaction capacity. Interestingly, in the yeast two-hybrid assays, none of the tomato FUL-like proteins could homodimerize, while Arabidopsis FUL homodimerizes both in EMSA and yeast two-hybrid assays (Figures 1A and 2A).

Given that the FUL–SOC1 complex in Arabidopsis regulates the transition to flowering, while the FUL–AG–SEP complex is involved in fruit development, we predicted that the tomato FUL2 protein would be able to largely complement the Arabidopsis *ful* mutant, while FUL1 and MBP20 would only be able to rescue the floral transition phenotype. To investigate this and thereby further test whether the subfunctionalized tomato *FUL*-like genes could be used to uncouple the functions of Arabidopsis *FUL*, we generated two types of transgenic lines. Because the flowering time phenotype of Arabidopsis *ful* mutants is mild (6), *35S:thFUL* lines were generated in the Col-0 background to get a clear read-out of the capacity to regulate flowering time (*thFUL*, tomato homolog of *FUL*, being *FUL1, FUL2*, *MBP10* or *MBP20*). Overexpression of *MBP20*, *FUL1*, *FUL2* and *FUL* in a WT background yielded transgenic lines with very early flowering, among lines with milder effects (Figure 2B), illustrating the capacity of these four genes to influence flowering time in a similar way in Arabidopsis.

In the second set of transgenic lines, the *ful-*7 (27) knock-out mutant was complemented with the tomato *FUL*-like genes and Arabidopsis FUL, each with the Arabidopsis *FUL* promoter and terminator (*pFUL:(th)FUL:tFUL*) to assess rescue of the mutant short silique phenotype (Figure 2C). Interestingly, Arabidopsis *FUL* was able to rescue the fruit phenotype almost completely, while *FUL2* was the only tomato co-ortholog that could partially rescue the phenotype. Complementation with *FUL1*, *MBP10* and *MBP20* resulted in siliques that were comparable to those of the *ful-7* mutant. Thus, the inability of FUL1 and MBP20 to interact with AG is probably impairing their function in the Arabidopsis fruit, while their function in flowering, linked to the interaction with SOC1, is similar to that of Arabidopsis FUL.

We questioned whether the difference between the complementation capacity of the FUL and FUL2 proteins could be explained by the fact that Arabidopsis FUL can form homodimers, while tomato FUL2 cannot. First, we confirmed that FUL2 cannot homodimerize even at very low concentrations of the competitive inhibitor 3-amino-1,2,4-triazole (3-AT) (Figure 2D and Supplementary Figure S6). To further investigate whether the FUL–FUL complex could be involved in silique development in addition to FUL-AG-SEP, we performed the DAP-seq experiment also with FUL-FUL. This yielded 2589 significantly enriched loci (*P*< 0.05), of which a large number was shared with the other complexes (Figure 2E). Interestingly, a set of 537 loci was significantly bound by FUL-FUL but not significantly by FUL-SOC1 or FUL-AG-SEP. Overlapping this list with differentially expressed genes in the *ful* mutant silique (18) led to the discovery of the carpel development gene HECATE 3 (HEC3) as a prominent target of the FUL-FUL homodimer (Figure 2F). Also, SPATULA (SPT), an interaction partner of HECs during carpel development, was more prominently bound by the FUL homodimer than by FUL-SOC1 or FUL-AG-SEP (Figure 2F). SPT and HEC1-3 function together to regulate carpel fusion and gynoecium development (63), and the results thus indicate that FUL homodimerization may be important for silique development. We investigated the nature of the CArG-box in the sequence under the HEC3 peak and discovered that it harbors two atypical CArG-boxes at 30 bp distance of each other, which contain instead of the canonical CArG-box (CC[A/T]_6_GG), CC[A/T]_6_CG or CC[A/T]_6_GC (Figure 2G). Thus, it seems that FUL-FUL is able to bind to divergent CArG-boxes that are hardly bound by FUL-SOC1 or FUL-AG-SEP. This property may render the homodimer indispensable for Arabidopsis silique development. Overall, our data indicate that FUL’s silique function depends both on homodimer formation and on interaction with AG-SEP, and that uncoupling of the flowering and fruit functions is possible if amino acid motif(s) can be identified that result in loss of interaction with AG/SEP and/or homodimerization, while retaining the interaction with SOC1.

### The loss of tomato FUL1 interaction with TAG1/AG is caused by a 2-aa polymorphism

**Figure 3.**
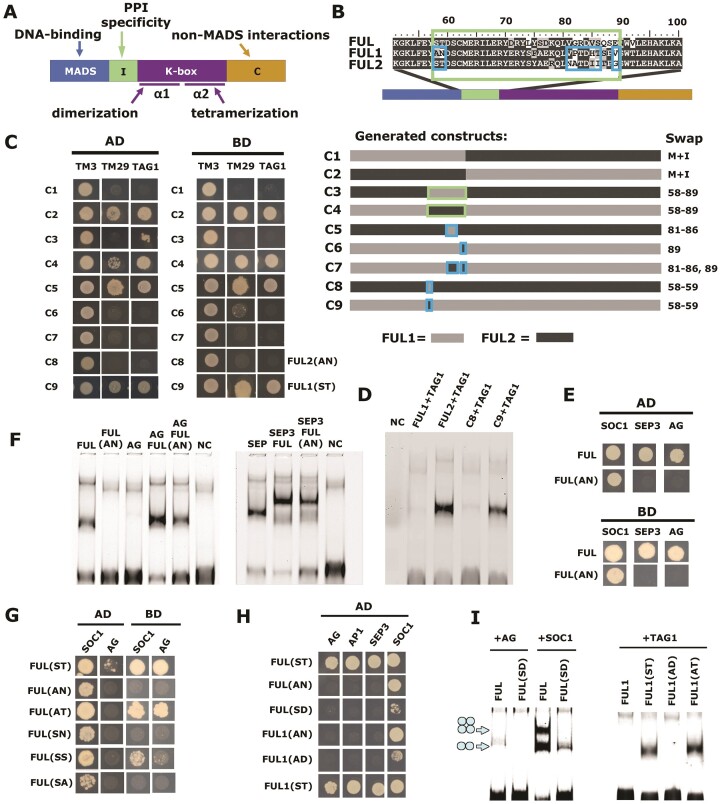
The loss of tomato FUL1 interaction with TAG1/AG is caused by a 2-aa polymorphism in the I-domain. (**A**) Schematic representation of the structure of MADS-domain TFs. (**B**) Schematic representation of the different constructs that were generated. The large box indicates the entire I-domain; smaller boxes inside indicate divergent motifs within the I-domain that have been swapped. (**C**) Yeast two-hybrid results of baits containing the different constructs with preys of TM3, TM29 or TAG1 (left) or vice versa (right). (**D**) EMSA showing that swapping of the AN/ST polymorphism results in a gain or loss of binding to a CArG-box containing probe, probably caused by gain/loss of interaction with TAG1. (**E**) Yeast two-hybrid results showing that also Arabidopsis FUL loses its interaction with AG if ST is changed for AN at positions 58–59. (**F**) EMSA results showing that homodimers and heterodimers of FUL(AN) bind less strongly or not at all (Supplementary Figure S6) to a CArG-box containing probe. NC = negative control (probe with only TNT reaction mixture added). (**G, H**) Yeast two-hybrid data showing the interaction of modified FUL proteins with SOC1, AG, AP1 and SEP3. (**I**) EMSA to test the capacity of different FUL and FUL1 versions to interact with AG, SOC1 or TAG1, and bind to a CArG-box containing probe.

We reasoned that identification of the amino acids causal for the different interaction capacities of FUL1 and FUL2 (i.e. with AG/SEP3) may be relatively easy, given the high sequence similarity between FUL1 and FUL2 (Supplementary Figure S5). Because the intervening (I) domain of MIKC-type proteins is important for protein–protein interaction specificity (Figure 3A) (64,65), we swapped protein domains in FUL1 and FUL2 and assessed the effect on the interactions with TM3, TM29 and TAG1, tomato homologs of SOC1, SEP3 and AG, respectively (Figure 3B, C). First, we confirmed that the I-region was determinant for the observed interaction pattern (constructs C1-C4). Then, we swapped smaller motifs ranging from one to six amino acids, based on sequence divergence between FUL1 and FUL2 (Supplementary Figure S5, Figure 3B; constructs C5-C9), and discovered that a 2-amino acid motif at the border of the M- and I domains (Figure 3C) is required for the interaction with TAG1. This motif is positioned at amino acids 58 and 59 and contains in FUL1 an alanine and asparagine (AN), while it consists of a serine and threonine (ST) in FUL2. FUL1 acquires the ability to interact with TAG1 if only AN is swapped for ST (Figure 3B, C).

To confirm these results, we performed an EMSA experiment with *in vitro* produced FUL1, FUL2, C8 (FUL1(ST)), C9 (FUL2(AN)) and TAG1 (Figure 3D). Also here, FUL1(ST) acquired the ability to interact with TAG1, while FUL2(AN) lost this ability. Next, we investigated whether changing the corresponding amino acids in Arabidopsis FUL would exert the same effect and abolish the interaction of FUL with AG and SEP3, while retaining the interaction with SOC1. FUL(AN) retained the interaction with SOC1, but lost the interaction with SEP3 and AG (Figure 3E). An EMSA showed similar results, although weak interaction of FUL(AN) with AG and/or SEP3 was observed in some EMSAs (Figure 3F), but not in others (Supplementary Figures S6 and S7). To determine whether Ser58 and Thr59 contributed equally to the interaction with AG, we performed yeast two-hybrid experiments with FUL(ST) (wild type), FUL(AN), FUL(AT), FUL(SN), FUL(SS) or FUL(SA) (Figure 3G and Supplementary Figure S8). Interestingly, the interaction with AG was only retained if Thr59 was present, regardless of whether there was an A or S at position 58. However, Ser58 contributes to the interaction strength, as yeast growth was less for FUL(AT) compared to FUL(ST). Similarly, tomato FUL1(AT) was perfectly capable of interacting with AG, AP1 and SEP3, similar to FUL1(ST) (Figure 3H), but yeast growth was less for the interaction with AG (Supplementary Figure S8). Testing the different FUL combinations against a larger set of Arabidopsis MADS-domain proteins also revealed a major difference between FUL(SA)/FUL(SN) and FUL(SS), where only the latter can interact with many Arabidopsis MADS-domain proteins, similar to FUL(AT/ST) (Supplementary Figures S9 and S10). Thus, while FUL(SS) specifically loses the interaction with AG, it retains the interaction with many others.

The discovery that it is important to have a Thr or Ser at position 59 made us hypothesize that phosphorylation may be involved. To test this, we designed phosphomimetic versions of FUL and FUL1, in which an aspartic acid (Asp; D) was introduced at position 59 instead of Thr or Asn, respectively, to mimic phosphorylated Thr (66). However, this resulted in a dramatic loss of overall interaction capacity, including the interaction with SOC1, which was highly reduced (Figure 3H and Supplementary Figure S8). EMSA experiments also showed that FUL and FUL1 are unable to interact with (tomato) AG if Thr59 is replaced by Asp59, and that the interaction with SOC1 is weakened (Figure 3I), thus not providing evidence for involvement of phosphorylation. These results are in line with data from Mengler et al. (67), who did not detect phosphorylated sites in FUL. Thus, Thr59 is crucial for the interactions of FUL with its MADS-domain partners, except for the interaction with SOC1, which tolerates other residues at position 59. Thr59 can be replaced by Ser59 but that results in the specific loss of AG interaction.

### The biological activity of FUL in the silique is reduced if interaction with AG/SEP is compromised

**Figure 4.**
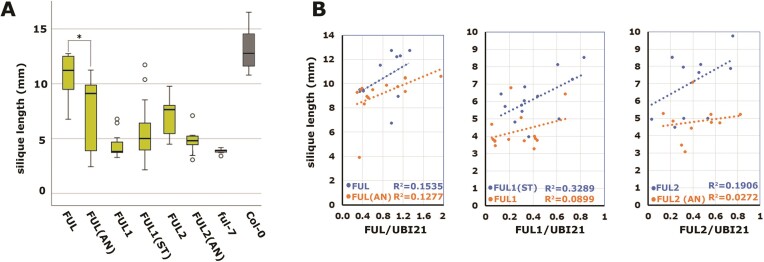
The biological activity of FUL, FUL1 and FUL2 depends on their interaction capacity. (**A**) Average silique lengths of the different pFUL:(th)FUL:tFUL transgenic lines in the *ful-7* mutant background. Each bar represents the data of at least 15 different transgenic lines. FUL is pFUL:FUL:tFUL, FUL(AN) is pFUL:FUL(AN):tFUL, etc. The two bars at the right indicate the wild-type and *ful* mutant controls. The asterisk indicates a significant difference between FUL(AN) and FUL lines (*P*< 0.01), as determined with one-way ANOVA followed by Tukey's post-hoc test. For this experiment, the seedlings were first selected on plates and then transferred to rockwool, resulting in plants with slightly smaller siliques compared to the experiment shown in Figure 2C. (**B**) Scatter plots showing the relationship between silique length (*y*-axis) and relative expression level (*x*-axis; ddCt (th)FUL/ddCtUBI21) for a subset of the same transgenic lines indicated in (**A**). The coefficient of determination (R2) is also included for each linear trendline. For each transgene combination, the orange dots and corresponding lower line depict the transgenic lines with Ala58 Asn59, while the blue dots and corresponding upper line depicts the transgenic lines with Ser58 Thr59.

To test both the effect of loss and gain of interaction with AG/SEP on the biological role of FUL in the silique, we transformed the Arabidopsis *ful-7* mutant with pFUL:FUL(AN):tFUL, pFUL:FUL1(ST):tFUL and pFUL:FUL2(AN):tFUL, measured the average silique lengths of independent transgenic lines (>15 per construct) and compared the results with the corresponding pFUL:(th)FUL:tFUL lines. In line with our expectations, swapping ST to AN at positions 58–59 in both FUL and FUL2 reduced the overall capacity to complement the *ful-7* silique phenotype while introducing ST in FUL1 instead of AN enhanced it (Figure 4A). However, the phenotypic variation for the different independent transgenic lines was considerable, complicating statistical analysis. Only the difference between the transgenic lines with pFUL:FUL:tFUL and the corresponding pFUL:FUL(AN):tFUL lines was statistically significant. Because complementation capacity depends on transgene expression, we determined for each transgenic line the expression levels at stage 12–16 siliques (44) for at least eight lines and linked these to the observed complementation phenotypes (Figure 4B). For each combination (FUL versus FUL(AN), FUL1 versus FUL1(ST), FUL2 versus FUL2(AN), constructs that encoded proteins with Ser58 Thr59 showed a steeper slope and better correlation between expression level and complementation capacity, indicating a higher functionality of these proteins in the silique. Remarkably, FUL1 and FUL2(AN) lines could complement *ful* siliques to a much lesser extent than Arabidopsis FUL(AN), although FUL(AN) and FUL2(AN) show a similar loss of interaction with AG-SEP and loss of homodimerization *in vitro* (Figure 3C–H and Supplementary Figures S6 and S7). To test whether minor differences exist between FUL(AN) and tomato FUL2(AN)/FUL1 in their capacity to interact with AG-SEP or to homodimerize, we performed yeast two-hybrid experiments at different concentrations of the competitive inhibitor 3-amino-1,2,4-triazole (3-AT) and performed additional EMSA experiments (Supplementary Figures S11 and S12). In the yeast experiments, FUL(AN) failed to interact with FUL or FUL(AN), SEP, and AG, even at low concentrations of 3-AT, similar to FUL2(AN), suggesting that the loss of interaction is equally strong for both proteins (Supplementary Figure S11). In EMSA, however, we did observe a more intense shift for FUL(AN)-AG compared to FUL2(AN)-AG (Supplementary Figure S12), suggesting that, depending on the conditions and/or the presence of DNA, FUL(AN) may have a better capacity to interact with other MADS-domain proteins than FUL2(AN) or FUL1(ST), which may explain its better functionality in the silique. Taken together, we show here that it is possible to biologically uncouple the functions of a multifunctional TF by modifying amino acids involved in specific protein–protein interactions, but biological complexity makes the interpretation *in planta* less straightforward. Nevertheless, the mild effect observed here could be very relevant in agriculture, for example in preventing pod shattering in Brassica (68) without affecting flowering time.

### The identified polymorphism is more widely important for specificity of MADS-domain protein–protein interactions

**Figure 5.**
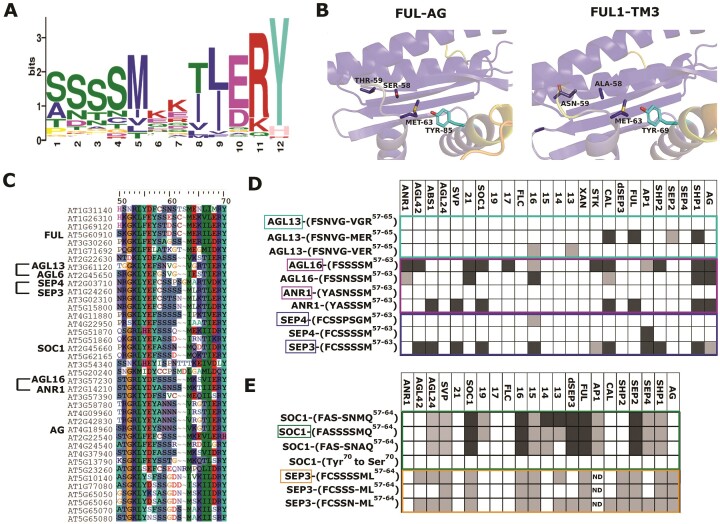
The identified polymorphism is more widely important for specificity of MADS-domain protein–protein interactions. (**A**) Most abundant motif in the I-region, identified by MEME based on 11966 MIKC-type proteins. Positions 1 and 2 of the sequence logo correspond to amino acid positions 58 and 59 in the MADS-domain proteins (**B**) Representative Alphafold2 dimers for FUL-AG and FUL1-TM3 showing the location of the AN and ST residues important for dimerization specificity. The proteins are color-coded by probability, from blue (higher probability) to yellow (lower probability). The residues lie in the loop between the M- and I-domains and do not directly contribute to the dimerization interface. Methionine and tyrosine residues putatively important for dimerization stability and/or specificity are shown, with the partner monomer tyrosine residue colored in cyan. Residues are shown as sticks, labeled and color-coded by atom. (**C**) Alignment of Arabidopsis MADS-domain TFs, showing the part of the protein that contains the identified motif. Proteins that were used to test the importance of the motif are indicated. (**D, E**) Constructs generated to test the Met63/Tyr70/loop hypothesis. The protein names indicated in boxes represent the wild-type sequence motifs; the others are swaps or modifications. Each column indicates a MADS-domain protein against which interaction was tested with yeast two-hybrid. See for details Supplementary Figures S18–S23. A dark box represents interactions in both directions (AD versus BD and BD versus AD); light boxes interactions in only one direction; white boxes no interaction; ND = not determined. The SEP3 constructs in (**E**) were only tested in one direction because of autoactivation of BD-SEP3. Where the non-mutated SEP3 was used, we made use of a dSEP3 version, which lacks part of the C-terminus and does not exhibit autoactivation. Number 21 indicates AGL21; 19 indicates AGL19, etc. XAN=AGL12(XAL1).

The polymorphism important for FUL interaction specificity (Ser58Thr59/Ala58Asn59) is located at the border of the M- and I- domains, a region previously associated with MADS-MADS dimerization specificity (65). To evaluate the general importance of this region for interaction specificity, we searched for motifs in the I-domain that were either generally conserved, or clade specific. The I-regions were extracted from a total of 11966 MIKC-type protein sequences from 1006 different plant species and motif mining and counting were conducted using MEME (48) and FIMO (49). A list of 18 motifs was identified, which were mostly clade or lineage specific (Supplementary Figure S13). Interestingly, the most abundant, generally conserved motif includes positions 58 and 59 at the start (Figure 5A). Two Ser residues are most common at these positions, but several alternative residues occur at relatively high frequencies, including Ala58 and Asn59. Several other residues in the motif, such as Met62, Glu67, Arg68 and Tyr69 (exact position dependent on protein) stand out as highly conserved. To obtain a structural explanation for the putative general importance of this motif for protein–protein interaction specificity, predictions of homo- and heterodimers for different combinations of FUL, AG, SEP3, FUL1, FUL2, TAG1 and TM3 were performed using Alphafold2 (AF2) (69). In addition, we calculated buried surface area and free energy of the resulting structures using PISA (54) (Supplementary Figure S14). The predicted structures are consistent with experimentally determined structures of the SEP3 DNA-binding domain (64) and the K-domain of SEP3 and SEP3-AG (70–72). Free energy calculations predicted that FUL, AG, TM3, FUL1 and FUL2 homodimers were less stable than heterodimers, whereas SOC1 homodimer scores are higher, consistent with its stable homodimerization formation. Residues 58–59 (AN/ST) are located in a lower probability flexible region between the C-terminal beta strand of the MADS domain and the N-terminal alpha helix of the I-domain and are not predicted to make direct contacts with the partner monomer at the dimerization interface (Figure 5B). This suggests that more subtle allosteric effects, which are poorly predicted by AF2 models, are important to confer dimerization specificity.

In the I-domain dimerization helix, the main interaction between the two monomers of a dimer pair is formed between Tyr70 (monomer A) and Met63 (monomer B). This is a favorable methionine–aromatic interaction, conserved in all interaction partners, except for AG. Figure 5B shows that Thr59 is not directly affecting the Met-Tyr pair, thus not providing an easy explanation for its effect on dimerization specificity. Positions 58–59 are in a loop region adjacent to the dimerization helix (positions 58–62, neighboring Met63). Given the difficulty to predict the structure of loop regions, we hypothesize that amino acid changes in the loop may have subtle impact on the Met–Tyr interaction at the dimer interface, thereby destabilizing/stabilizing this interaction and allowing only specific interactions. The fact that AG has Ala63 instead of Met63 may explain why FUL-AG requires Thr59, while the other interactions also tolerate Ser59. Interestingly, SOC1 has a different loop sequence, which is one amino acid shorter, and may act as a compensation mechanism for the Thr59/Asn59 FUL mutation, stabilizing the dimer interface through a more rigid loop or a different loop conformation. As AF2 structural predictions do not yet allow unambiguous computational predictions of dimerization specificity and interaction patterns for the MADS-domain TFs, we set off to experimentally test the loop-Met-Tyr hypothesis. Within the Arabidopsis MIKC-type proteins, we selected paralogs/close homologs with diverged loop residues and different interaction profiles (55): AGL16/ANR1, AGL16/AGL13 and SEP3/SEP4 (Figure 5C). Constructs were designed in which one or a few amino acids were swapped with that of the close homolog (Figure 5D and Supplementary Figures S15–S17). In the case of the AGL16/ANR1 pair, swapping amino acids drastically changed the interaction capacity, with ANR1 gaining many interactions, while AGL16 lost most of them. A gain of interactions was also observed for the mutated AGL13 protein, in particular when also Met63 was introduced instead of Val63. Only SEP4 did not display any additional interactions upon introduction of amino acids from the SEP3 loop, but this may not be surprising given the high number of amino acid differences between the SEP3 and SEP4 K-domains (Supplementary Figure S17).

To further test loop length and Met63 and Tyr70 importance, we generated additional mutation constructs with changes in the SEP3 and SOC1 proteins (Supplementary Figures S18–S19). Extending the loop in SOC1 by one amino acid (Ser60) clearly had an effect on several specific interactions, with a loss of interaction of BD-SOC1_S59-62 with AD-AGL13 and AD-AGL14, and weakening of the interaction with AD-SEP2. Interestingly, a version of SOC1 with regular short loop length, but Ala63 instead of Met63, affected the same interactions, with an additional loss of the interaction of AD-SOC1_Ala63 with AGL19 (Figure 5E and Supplementary Figures S19–S23). Thus, it seems that either extending loop length or replacing Met63 results in reduced interaction affinities with a specific subset of SOC1 interactors. The most drastic effect on interaction capacity was obtained by changing Tyr70 to Ser70 in SOC1. This abolished practically all interactions, indicating that Tyr70 is indeed a crucial residue for the MADS-domain protein dimerization (Figure 5E). We constructed two different SEP3 versions to test whether shortening of the loop in SEP3 could conversely lead to gains in interaction, and observed additional interactions of AD-SEP3_S59S60N61 (representing the exact same loop sequence as in SOC1) with BD-SEP3, BD-SEP4 and BD-CAL. However, the clone AD-SEP3_S59S60S61 did not gain these interactions, but rather lost a few, suggesting that the Asn61 in SOC1 is important in addition to the short loop (Figure 5E and Supplementary Figures S19–S23).

Altogether, we used here the tomato subfunctionalized co-orthologs FUL1 and FUL2 to pinpoint residues that are crucial for protein–protein interaction specificity, and by investigating these further, we identified a protein motif that is widely important for the protein–protein interaction specificity of MADS-domain proteins. The stabilization of the Met–Tyr interaction at the dimer interface depends on the composition of the adjacent loop, which allows for only a few specific, or many dimerization partners.

## Discussion

Here, we used the tomato FUL co-orthologs FUL1 and FUL2, which have, to some extent, subfunctionalized to gain insight into the different functions of Arabidopsis FUL on the protein level. We discovered that depending on the tissue-specific complexes formed by FUL in the inflorescence or silique, there are differences in the target gene sets to which FUL is binding. By linking a polymorphism in the tomato FUL1 and FUL2 sequences to variation in their interaction patterns, we identified a 2-amino acid motif that determines the ability to interact with AG and SEP, while it did not affect interactions with SOC1. By identifying this motif, we gained additional evidence that the most important region for MADS–MADS interaction specificity is the I-domain, as was previously suggested (63,64), and formulated a hypothesis on how the motif, located at the border of the M- and I-domains, may structurally influence interaction affinity. We showed that the motif is also important for several other MADS–MADS interactions, but it is not the only determinant of specificity. Thus, further studies will have to add pieces to the puzzle to get to a more comprehensive understanding of the determinants of interaction specificity. Modifying the two-amino acid motif in the multifunctional Arabidopsis FUL protein specifically reduced its functionality in the fruit, thereby allowing, at least partially, a specific functional modification *in planta*. However, the biological effect was milder than expected based on the *in vitro* experiments, illustrating that translation of *in vitro* results to the complex *in planta* situation is still a challenge. Nevertheless, quantitative differences, even relatively modest ones, are desired in crop breeding, and modifications in promoter regions have been successfully used to achieve desired quantitative effects (15,16). The ability to dissect functions at the protein level can additionally be highly valuable and represents a feasible method for applying protein engineering for crop improvement.

In addition to developing a new approach, the uncoupling of FUL’s inflorescence and silique functions also provided novel insights into complex-specific target gene regulation. Multifunctional proteins such as Arabidopsis FUL have a broad expression pattern and can interact with a plethora of other proteins to fulfil their functions in different plant tissues. However, within the context of each cell type, only a subset of interaction partners is present and, dependent on the cell-specific complexes that can be formed, different sets of target genes are regulated. That MADS-domain proteins regulate different target gene sets based on the cell-type specific complexes is the basis of the well-known ABCE-model of floral organ development, but it is still far from understood how this differentiation in target gene sets is achieved, given that all MADS-domain proteins bind CArG-boxes (20,73,74). Recent insights suggest that the CC and GG nucleotides in the CArG-box are essential for optimal contact between dimer and DNA in the major groove, while the A-tract in the middle of the sequence determines minor groove width, which is also determinant for MADS protein binding (22,23). It has been shown that SEP3 binds DNA with an A-tract and narrow minor groove with higher affinity, a feature that depends on a highly conserved arginine residue at the N-terminus of MADS-domain proteins, which extends into the minor groove (22). The fact that AG does not have this arginine may suggest that it has other DNA shape requirements. The importance of the additional A/T-stretches 5′ and 3′ of the canonical CArG-box for AG binding (Figure 1H), may be related to this. In addition to DNA base- and shape readout, the affinity for tetramers to bind two CArG-boxes likely depends on their orientation and distance from each other (22–24). Finally, the recruitment of complex-specific co-factors can affect DNA binding and target gene regulation. *In planta* pull-down of MADS–domain complexes showed that several co-factors, including co-repressors/activators and chromatin remodelers, are likely associated with MADS–domain complexes (17). Much is yet unknown about the function(s) of the much more variable C-terminal domain of MADS-domain proteins. It is not involved in DNA-binding, but likely interacts with a variety of co-factors for specific activation or repression of target gene expression, thereby contributing to complex-specific regulation (23,74). Possibly, the better functionality of Arabidopsis FUL in the silique compared to tomato FUL2 may also be caused by C-terminal-mediated-specific interactions (in addition to its ability to homodimerize). In conclusion, the target gene specificity of different MADS–domain complexes depends on a combination of features, and our DAP-seq approach has shed more light on complex-specific variation in the DNA binding motifs, which contribute to base and shape readout.

While most target genes are overlapping between the FUL–FUL, FUL–SOC1 and FUL–AG–SEP complexes, it may be the specific targets that determine tissue specification, probably *in planta* depending on interaction affinities and level of expression. For example, in the Arabidopsis meristem, SOC1 is higher expressed than FUL upon the transition to flowering (TraVa database (75)) and given the very stable FUL–SOC1 complex (Figure 1A and Supplementary Figure S7), a FUL–FUL complex may not exist in this tissue. However, *SOC1* is not expressed in the gynoecium/silique, and it is probably the FUL–FUL complex, present in the center of the floral meristem from stage three onwards (76), which specifically represses the *SPT/HEC* genes at a certain stage where other high-affinity interactors (e.g. SEP3) are less abundant. The patterning of the Arabidopsis silique is tightly regulated to ensure the development of a dehiscence zone (77,78), but the tomato fruit does not need to be patterned, as shattering does not take place to release the seeds. The inability of tomato FUL1 and FUL2 to homodimerize may well be correlated with this lost requirement for patterning. Tomato FUL2 does have an early function in fruit growth however, while FUL1 does not (33). This function of FUL2 may depend on its interaction with TAG1, which is expressed at this stage (Tomato Expression Atlas (79)), while FUL1 becomes only expressed later during the onset of fruit ripening, in line with its inability to interact with TAG1. Interestingly, FUL1 and FUL2 both interact with the SEP3 ortholog RIPENING INHIBITOR (RIN) during the onset of fruit ripening (80), while FUL1 is not able to interact with Arabidopsis SEP3. Thus, co-evolution of FUL1 and RIN ensured their specific interactions, and FUL1 has less subfunctionalized than would be expected based on the heterologous experiments in Arabidopsis. Linking protein sequence divergence of MADS-domain proteins to divergence in protein–protein interaction profiles and functional traits is still a challenge, but our discovery of the Met/Tyr/loop-motif as a more general actor in MADS-domain protein–protein interaction specificity will facilitate future attempts for the MADS-domain family.

## Supplementary Material

gkae963_Supplemental_Files

## Data Availability

The DAP-seq data have been deposited in the GEO database under accession GSE263113.

## Notes

Present address: Aalt D.J. van Dijk, Biosystems Data Analysis, Swammerdam Institute for Life Sciences, University of Amsterdam, Amsterdam, the Netherlands.

Present address: Miguel Correa Marrero, Department of Computational Systems Biology, Institute of Biology, ETH Zurich, Otto-Stern-Weg 3, 8093 Zurich, Switzerland; Swiss Institute of Bioinformatics, 1015 Lausanne, Switzerland.

Present address: Marian Bemer, Biosystematics Group, Wageningen University & Research, Wageningen 6708 PB, the Netherlands.
