# Supplementary material for: Uncoupling FRUITFULL’s functions through modification of a protein motif identified by co-ortholog analysis
Source: Nucleic Acids Res. 2024 Oct 30;52(21):13290–304. doi: 10.1093/nar/gkae963 (PMC11602133; doi:10.1093/nar/gkae963)
Supplement: gkae963_Supplemental_Files [file gkae963_supplemental_files.zip › Supplementary Figures.pdf]

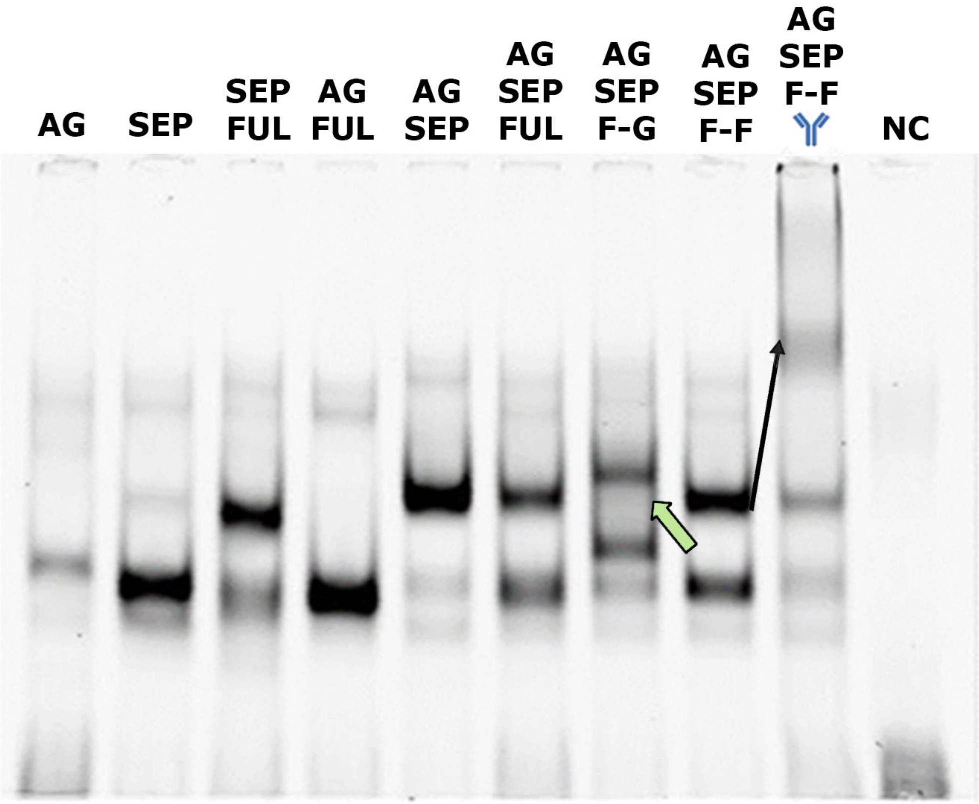

Figure S1. EMSA using the SAUR10 probe with a single canonical CArG-box (27) and different combinations of the FUL*, SEP3 and AG proteins*. *The blue Y indicates the addition of the FLAG antibody, the green arrow indicates the increased size of the FUL-GFP complexes (mainly FUL-AG and FUL-AG-SEP3); the black arrow shows the shifted complexes that contain FUL-linker-FLAG***.** Abbreviations: *SOC, SOC1; SEP, SEP3; F-G, FUL-linker-GFP; F-F, FUL-linker-FLAG; NC, negative control (probe with only TNT reaction mixture)*

GO biological processes, FDR P < 0.05

GO biological process complete	Arabidopsis thaliana (REF) #	upload_1 (▼ Hierarchy NEW!) #	expected	Fold Enrichment	+/-	raw P value	FDR
transmitting tissue development	4	3	.25	12.21	+	8.83E-04	5.00E-02
↳floral whorl development	219	30	13.45	2.23	+	4.61E-05	4.82E-03
↳flower development	378	46	23.22	1.98	+	1.12E-05	1.73E-03
↳reproductive shoot system development	396	46	24.33	1.89	+	3.21E-05	3.56E-03
↳post-embryonic development	1573	134	96.64	1.39	+	1.18E-04	9.45E-03
↳multicellular organism development	2643	210	162.38	1.29	+	8.65E-05	7.73E-03
↳multicellular organismal process	2923	240	179.58	1.34	+	2.17E-06	4.30E-04
↳anatomical structure development	3088	264	189.72	1.39	+	1.83E-08	5.64E-06
↳developmental process	3192	267	196.11	1.36	+	9.10E-08	2.52E-05
↳reproductive structure development	1278	110	78.52	1.40	+	3.32E-04	2.27E-02
↳reproductive system development	1280	110	78.64	1.40	+	3.39E-04	2.26E-02
↳system development	2033	170	124.90	1.36	+	3.49E-05	3.80E-03
↳developmental process involved in reproduction	1537	132	94.43	1.40	+	9.84E-05	8.27E-03
↳reproductive process	1820	154	111.82	1.38	+	5.05E-05	5.18E-03
↳shoot system development	791	83	48.60	1.71	+	1.83E-06	3.76E-04
carpel formation	4	3	.25	12.21	+	8.83E-04	4.95E-02
↳floral organ formation	25	8	1.54	5.21	+	8.40E-05	7.76E-03
↳floral organ morphogenesis	50	11	3.07	3.58	+	1.84E-04	1.34E-02
↳floral organ development	270	33	16.59	1.99	+	1.70E-04	1.29E-02
↳plant organ development	1148	101	70.53	1.43	+	2.61E-04	1.86E-02
↳post-embryonic plant organ morphogenesis	131	22	8.05	2.73	+	1.56E-05	2.06E-03
↳post-embryonic plant organ development	192	25	11.80	2.12	+	3.83E-04	2.47E-02
↳anatomical structure morphogenesis	990	95	60.82	1.56	+	1.48E-05	2.00E-03
↳post-embryonic plant morphogenesis	222	31	13.64	2.27	+	1.72E-05	2.17E-03
↳plant organ formation	118	19	7.25	2.62	+	1.04E-04	8.59E-03
↳phyllome development	539	57	33.11	1.72	+	6.04E-05	5.98E-03
positive regulation of anthocyanin biosynthetic process	4	3	.25	12.21	+	8.83E-04	4.90E-02
↳regulation of primary metabolic process	2611	252	160.41	1.57	+	2.27E-13	1.26E-10
↳regulation of metabolic process	3154	302	193.77	1.56	+	1.12E-15	1.55E-12
↳regulation of biological process	5374	449	330.17	1.36	+	4.28E-13	1.83E-10
↳biological regulation	5704	472	350.44	1.35	+	3.55E-13	1.79E-10
↳regulation of biosynthetic process	2781	280	170.86	1.64	+	3.11E-17	1.72E-13
↳positive regulation of anthocyanin metabolic process	6	4	.37	10.85	+	1.93E-04	1.39E-02
↳positive regulation of metabolic process	762	85	46.82	1.82	+	9.64E-08	2.54E-05
↳positive regulation of biological process	1239	120	76.12	1.58	+	8.00E-07	1.93E-04
↳positive regulation of biosynthetic process	625	68	38.40	1.77	+	4.49E-06	8.03E-04
glycerolipid catabolic process	7	4	.43	9.30	+	4.28E-04	2.69E-02
↳lipid metabolic process	935	85	57.44	1.48	+	2.98E-04	2.09E-02
floral meristem determinacy	21	7	1.29	5.43	+	1.77E-04	1.32E-02
↳meristem determinacy	25	8	1.54	5.21	+	8.40E-05	7.90E-03
↳meristem development	271	34	16.65	2.04	+	9.84E-05	8.39E-03
↳tissue development	687	77	42.21	1.82	+	3.12E-07	7.86E-05
plant-type cell wall cellulose metabolic process	19	6	1.17	5.14	+	7.22E-04	4.21E-02
↳cellulose metabolic process	62	13	3.81	3.41	+	8.30E-05	7.93E-03
↳beta-glucan metabolic process	75	16	4.61	3.47	+	1.03E-05	1.69E-03
positive regulation of abscisic acid-activated signaling pathway	53	13	3.26	3.99	+	1.41E-05	2.11E-03
↳positive regulation of signal transduction	98	18	6.02	2.99	+	2.63E-05	3.17E-03
↳positive regulation of response to stimulus	291	35	17.88	1.96	+	1.77E-04	1.31E-02
↳regulation of response to stimulus	847	78	52.04	1.50	+	3.51E-04	2.29E-02
↳positive regulation of signaling	99	18	6.08	2.96	+	3.03E-05	3.43E-03
↳positive regulation of cell communication	99	18	6.08	2.96	+	3.03E-05	3.50E-03
↳regulation of cellular process	4787	394	294.10	1.34	+	1.80E-10	6.66E-08
↳positive regulation of cellular process	897	92	55.11	1.67	+	1.31E-06	3.02E-04
↳regulation of abscisic acid-activated signaling pathway	132	20	8.11	2.47	+	1.63E-04	1.29E-02
↳regulation of cellular response to alcohol	132	20	8.11	2.47	+	1.63E-04	1.28E-02
↳regulation of response to alcohol	132	20	8.11	2.47	+	1.63E-04	1.26E-02
cellulose biosynthetic process	56	11	3.44	3.20	+	5.22E-04	3.15E-02
↳beta-glucan biosynthetic process	68	14	4.18	3.35	+	5.54E-05	5.58E-03
maintenance of meristem identity	73	13	4.48	2.90	+	4.59E-04	2.86E-02
response to gibberellin	136	21	8.36	2.51	+	8.60E-05	7.81E-03
↳response to lipid	892	97	54.80	1.77	+	5.15E-08	1.50E-05
↳response to chemical	2711	222	166.56	1.33	+	7.59E-06	1.28E-03
↳response to stimulus	5862	418	360.15	1.16	+	4.70E-04	2.90E-02
↳response to oxygen-containing compound	1575	144	96.76	1.49	+	1.38E-06	3.05E-04
↳response to hormone	1376	141	84.54	1.67	+	1.63E-09	5.64E-07
↳response to endogenous stimulus	1386	142	85.15	1.67	+	1.75E-09	5.69E-07
regulation of small molecule metabolic process	135	19	8.29	2.29	+	7.96E-04	4.60E-02
positive regulation of transcription by RNA polymerase II	170	23	10.44	2.20	+	3.30E-04	2.28E-02
↳positive regulation of DNA-templated transcription	475	56	29.18	1.92	+	2.82E-06	5.22E-04
↳regulation of DNA-templated transcription	2076	211	127.54	1.65	+	2.24E-13	1.38E-10
↳regulation of RNA biosynthetic process	2078	212	127.67	1.66	+	1.12E-13	7.76E-11
↳regulation of macromolecule biosynthetic process	2637	259	162.01	1.60	+	1.07E-14	1.18E-11
↳regulation of macromolecule metabolic process	2856	272	175.47	1.55	+	8.89E-14	7.04E-11
↳regulation of cellular biosynthetic process	2718	274	166.99	1.64	+	6.31E-17	1.75E-13
↳regulation of cellular metabolic process	2922	287	179.52	1.60	+	3.07E-16	5.68E-13
↳regulation of RNA metabolic process	2184	218	134.18	1.62	+	4.15E-13	1.92E-10
↳regulation of nucleobase-containing compound metabolic process	2273	224	139.65	1.60	+	7.72E-13	3.06E-10
↳regulation of gene expression	2598	255	159.62	1.60	+	1.96E-14	1.81E-11
↳positive regulation of RNA biosynthetic process	475	56	29.18	1.92	+	2.82E-06	5.40E-04
↳positive regulation of RNA metabolic process	539	60	33.11	1.81	+	1.07E-05	1.70E-03
↳positive regulation of nucleobase-containing compound metabolic process	560	62	34.41	1.80	+	7.10E-06	1.23E-03
↳positive regulation of macromolecule metabolic process	673	70	41.35	1.69	+	2.00E-05	2.46E-03
↳positive regulation of macromolecule biosynthetic process	563	60	34.59	1.73	+	3.76E-05	4.01E-03
↳positive regulation of cellular biosynthetic process	598	64	36.74	1.74	+	1.46E-05	2.02E-03
↳positive regulation of cellular metabolic process	637	71	39.14	1.81	+	1.46E-06	3.11E-04
↳regulation of transcription by RNA polymerase II	479	49	29.43	1.67	+	4.94E-04	3.01E-02
cell division	216	29	13.27	2.19	+	7.94E-05	7.72E-03
response to nitrogen compound	180	24	11.06	2.17	+	4.26E-04	2.71E-02
response to abscisic acid	532	59	32.68	1.81	+	1.44E-05	2.05E-03
↳response to alcohol	539	59	33.11	1.78	+	1.71E-05	2.20E-03
hormone-mediated signaling pathway	473	51	29.06	1.75	+	9.62E-05	8.33E-03
↳cellular response to hormone stimulus	558	58	34.28	1.69	+	1.11E-04	9.01E-03
↳cellular response to endogenous stimulus	569	59	34.96	1.69	+	9.02E-05	7.93E-03
cell growth	411	43	25.25	1.70	+	8.21E-04	4.69E-02
regulation of multicellular organismal process	505	52	31.03	1.68	+	3.35E-04	2.26E-02
negative regulation of cellular biosynthetic process	561	56	34.47	1.62	+	3.50E-04	2.31E-02
↳negative regulation of biosynthetic process	569	56	34.96	1.60	+	5.32E-04	3.14E-02
↳negative regulation of biological process	1156	108	71.02	1.52	+	1.42E-05	2.07E-03
↳negative regulation of cellular metabolic process	596	58	36.62	1.58	+	5.27E-04	3.14E-02
regulation of developmental process	875	85	53.76	1.58	+	2.98E-05	3.51E-03

Figure S2. GO-term enrichment analysis for the pooled DAP-seq loci with peaks at p>0.001, selecting biological processes. The TAIR GO-term enrichment tool was used (www.arabidopsis.org; https://pantherdb.org/webservices/go/overrep.jsp).

GO molecular function, FDR P < 0.05

	Arabidopsis thaliana (REF)	upload_1 (▼ Hierarchy NEW! ⓘ)					
GO molecular function complete	#	#	expected	Fold Enrichment	+/-	raw P value	FDR
RNA polymerase II cis-regulatory region sequence-specific DNA binding	271	36	16.65	2.16	+	1.68E-05	4.92E-03
↳cis-regulatory region sequence-specific DNA binding	324	44	19.91	2.21	+	9.48E-07	3.39E-04
↳transcription cis-regulatory region binding	1008	117	61.93	1.89	+	3.10E-11	2.50E-08
↳transcription regulatory region nucleic acid binding	1008	117	61.93	1.89	+	3.10E-11	3.33E-08
↳sequence-specific double-stranded DNA binding	1028	117	63.16	1.85	+	1.21E-10	7.77E-08
↳double-stranded DNA binding	1145	121	70.35	1.72	+	5.25E-09	2.41E-06
↳DNA binding	1849	173	113.60	1.52	+	2.20E-08	8.86E-06
↳sequence-specific DNA binding	1281	133	78.70	1.69	+	2.65E-09	1.42E-06
↳RNA polymerase II transcription regulatory region sequence-specific DNA binding	297	37	18.25	2.03	+	4.44E-05	1.19E-02
DNA-binding transcription factor activity, RNA polymerase II-specific	317	40	19.48	2.05	+	1.59E-05	5.12E-03
↳DNA-binding transcription factor activity	1680	189	103.22	1.83	+	2.87E-16	9.23E-13
↳transcription regulator activity	1828	198	112.31	1.76	+	2.79E-15	4.49E-12

Figure S3. GO-term enrichment analysis for the pooled DAP-seq loci with peaks at p>0.001, selecting molecular function. The TAIR GO-term enrichment tool was used (www.arabidopsis.org; https://pantherdb.org/webservices/go/overrep.jsp).

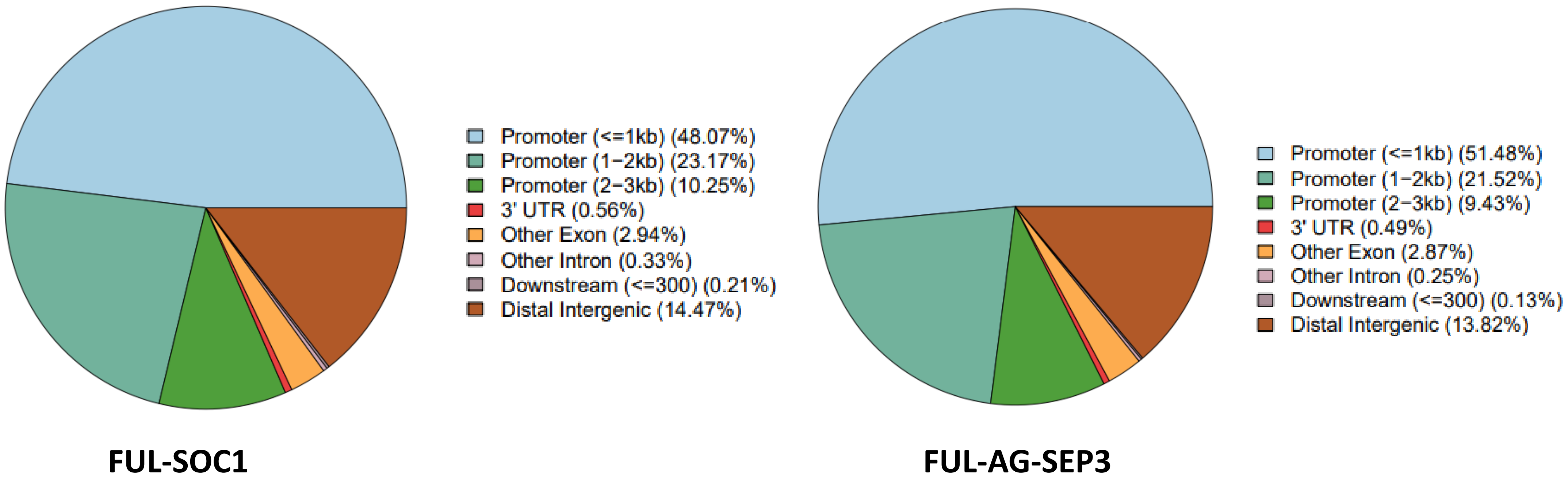

Figure S4. DAP results of the FUL-SOC1 and FUL-AG-SEP3 complexes. The binding events occur predominantly in the upstream regulatory region.

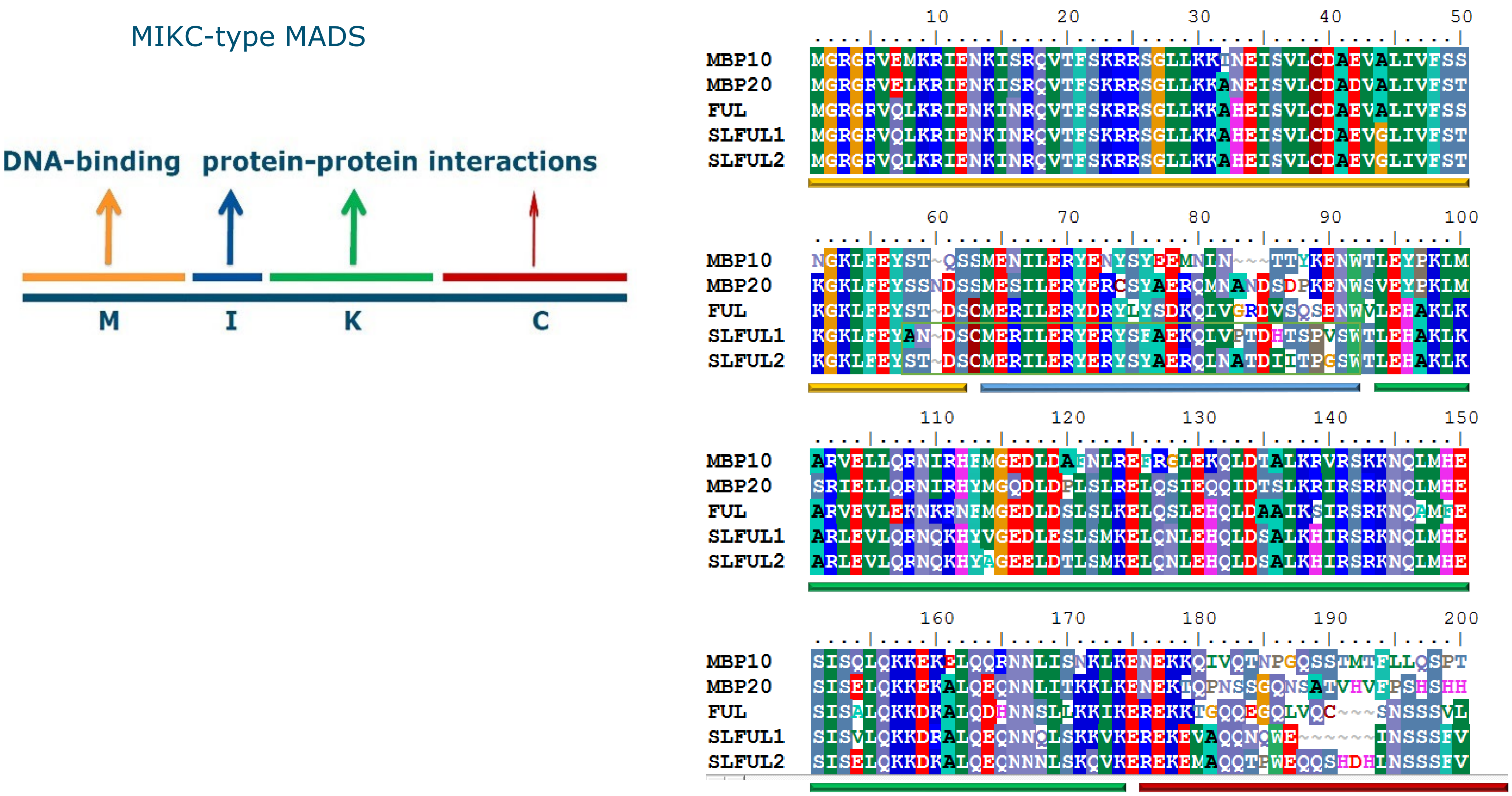

Figure S5. The protein sequences of Arabidopsis FUL and the tomato FUL-like proteins. The entire M-, I- and K-domains are displayed, as well as a part of the more divergent C-domains.

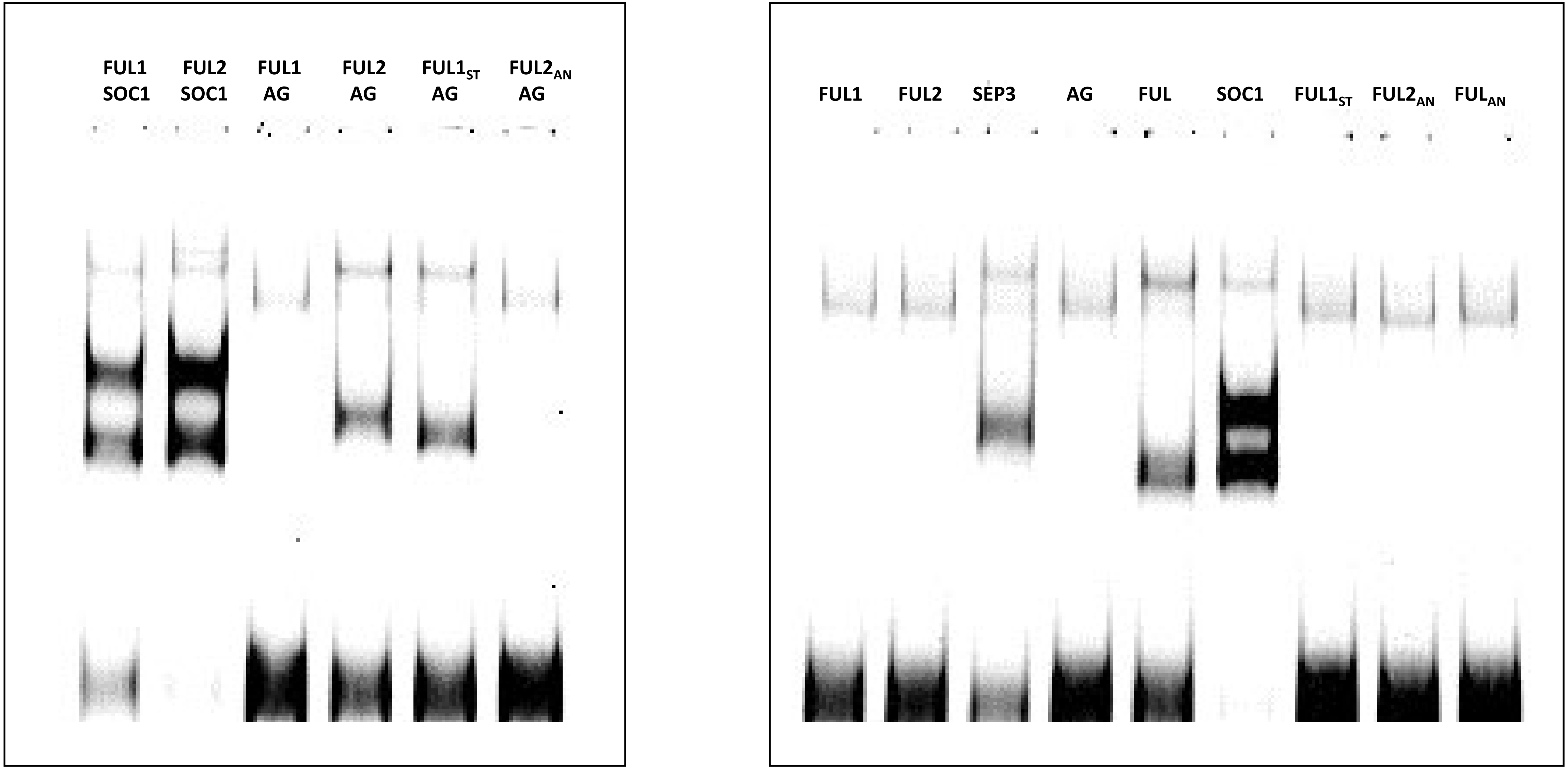

Figure S6. EMSA experiment showing the interaction capacity of the Arabidopsis and tomato FULs and their ST/AN variants. The canonical SAUR10 probe was used (Bemer et al., 2017).

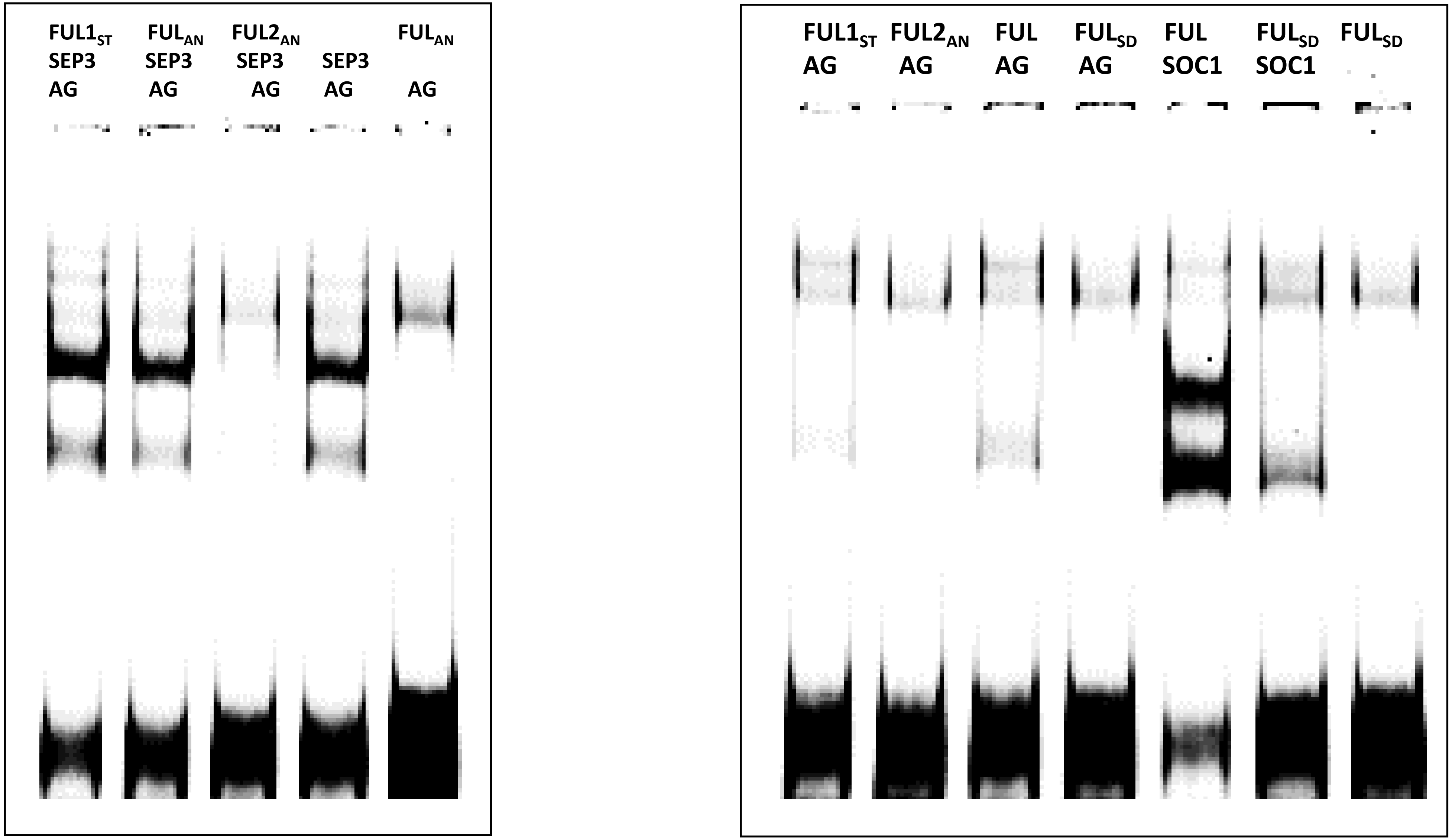

Figure S7. EMSA experiment showing the interaction capacity of the Arabidopsis and tomato FULs and some ST/AN/SD variants. The canonical *SAUR10* probe was used (Bemer et al., 2017).

AD/BD	SOC1	AG
FUL (ST)	1	1
FUL (AN)	0	0
FUL (AT)	1	1
FUL (SS)	1	1
FUL (SA)	0	0
FUL (SD)	0	0
SlFUL1 (AN)	1	0
SlFUL1 (AD)	0	0
SlFUL1 (AT)	1	1
SlFUL1 (ST)	1	1

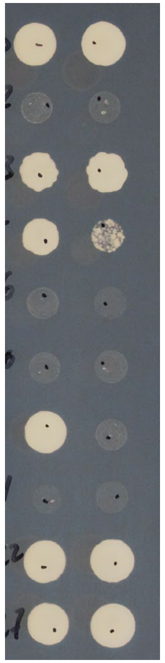

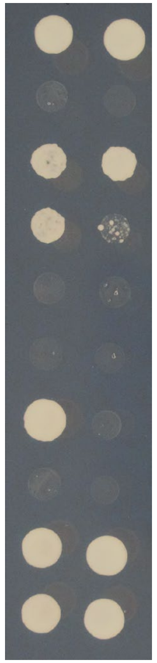

BD/AD	AG	AP1	SEP3	SOC1
FUL (ST)	1	1	1	1
FUL (AN)	0	0	0	1
FUL (AT)	0	1	1	1
FUL (SS)	0	1	1	1
FUL (SA)	0	0	0	1
FUL (SD)	0	0	0	1
SlFUL1 (AN)	0	0	0	1
SlFUL1 (AD)	0	0	0	1
SlFUL1 (AT)	1	1	1	1
SlFUL1 (ST)	1	1	1	1

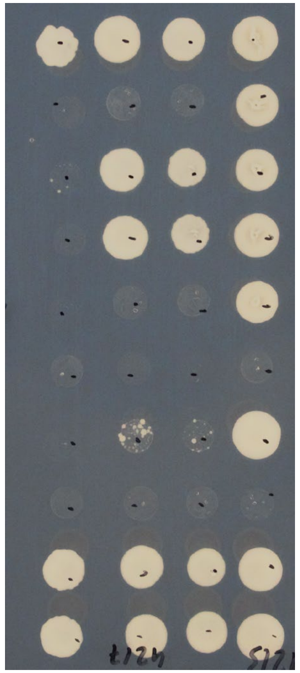

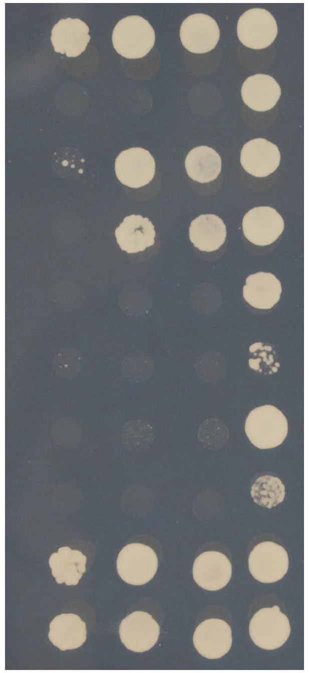

Figure S8. Yeast two-hybrid testing Arabidopsis FUL and tomato FUL1 with different residues on positions 58 and 59 against SOC1, AG, AP1 and SEP3. 5 mM 3AT was used for the screening and the plates were imaged after 3 days at 22 °C.

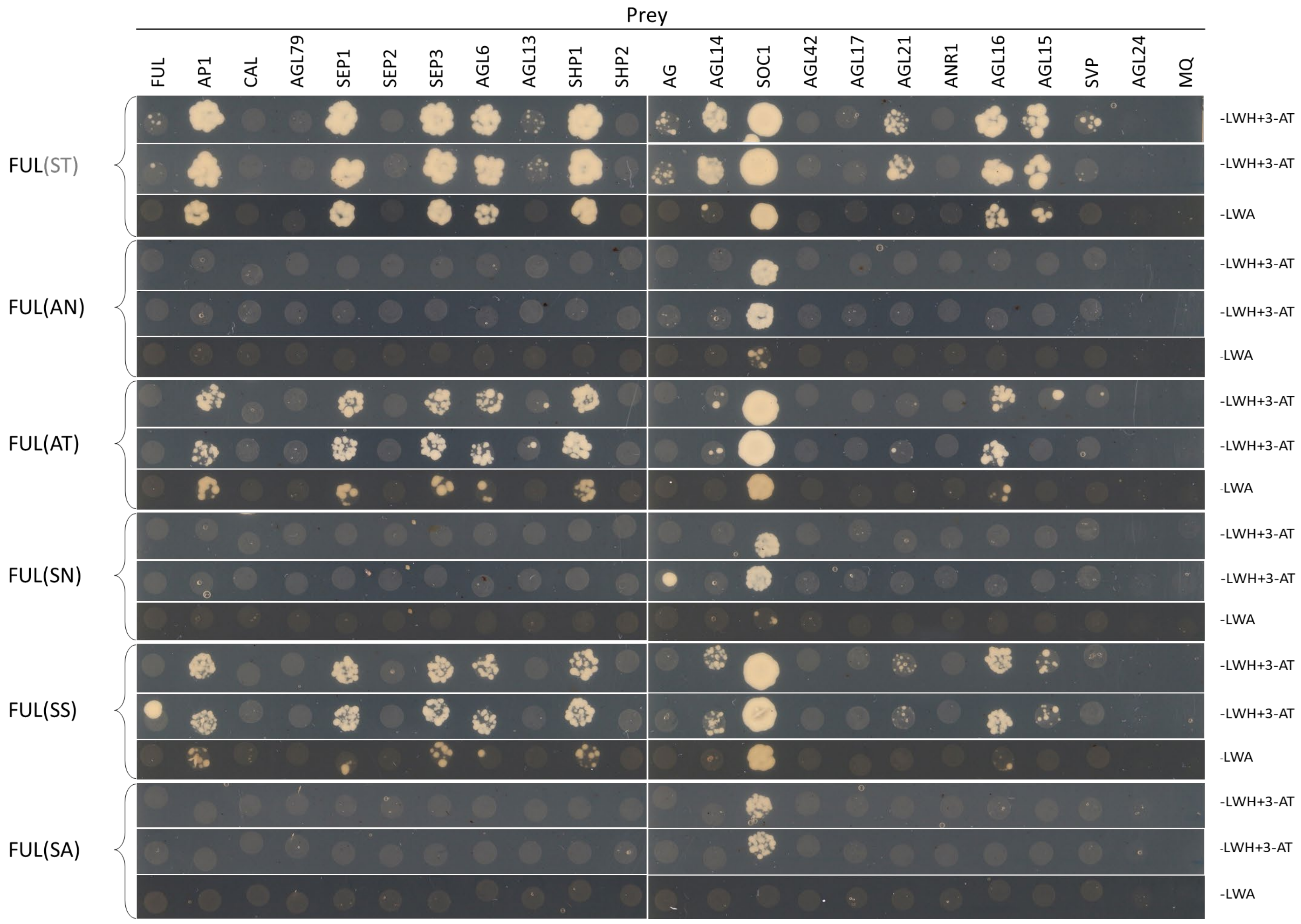

Figure S9. Yeast two-hybrid testing Arabidopsis FUL with different residues on positions 58 and 59 against a selected set of previously reported interactors (De Folter et al., 2011). FUL constructs as bait (fused to BD). For each combination, two replicates were spotted on -LWH with 5 mM 3-AT and one on –LWA (see labels on right side). The plates were imaged after 3 days at 20 °C.

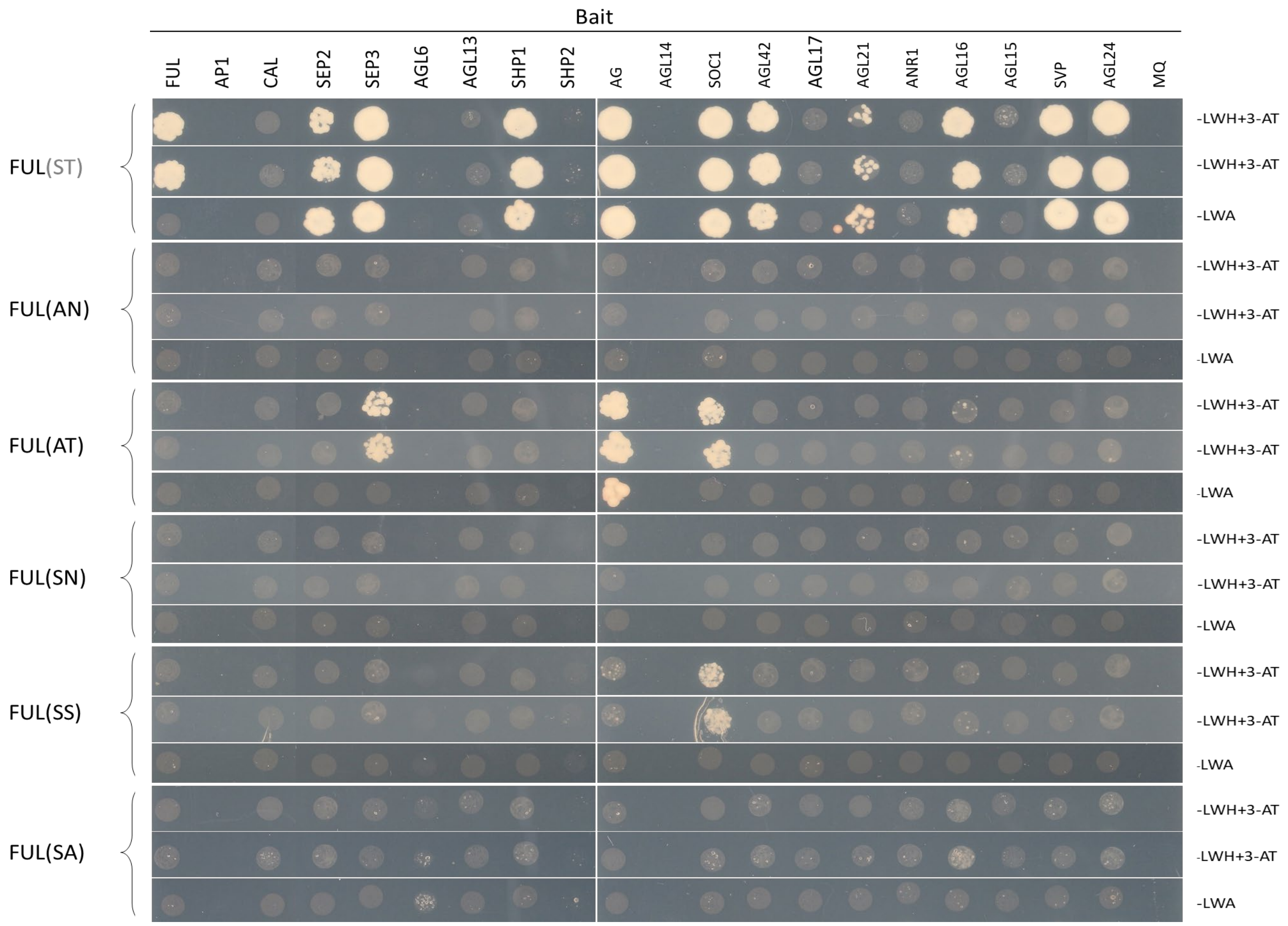

Figure S10. Yeast two-hybrid testing Arabidopsis FUL with different residues on positions 58 and 59 against a selected set of previously reported interactors (De Folter et al., 2011). FUL constructs as prey (fused to AD). For each combination, two replicates were spotted on -LWH with 5 mM 3-AT and one on –LWA (see labels on right side). The plates were imaged after 3 days at 20 °C.

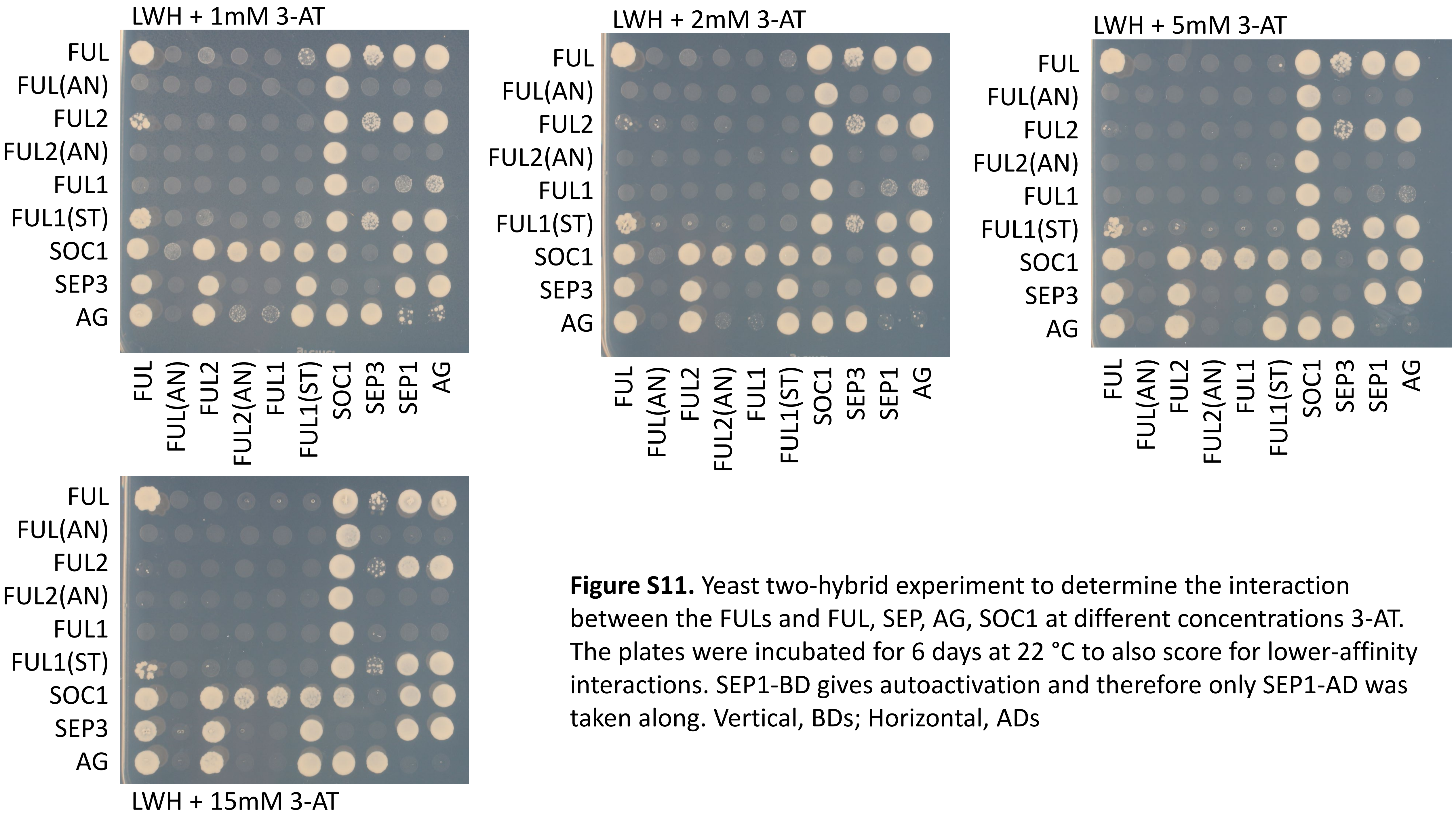

Figure S11. Yeast two-hybrid experiment to determine the interaction between the FULs and FUL, SEP, AG, SOC1 at different concentrations 3-AT. The plates were incubated for 6 days at 22 °C to also score for lower-affinity interactions. SEP1-BD gives autoactivation and therefore only SEP1-AD was taken along. Vertical, BDs; Horizontal, ADs

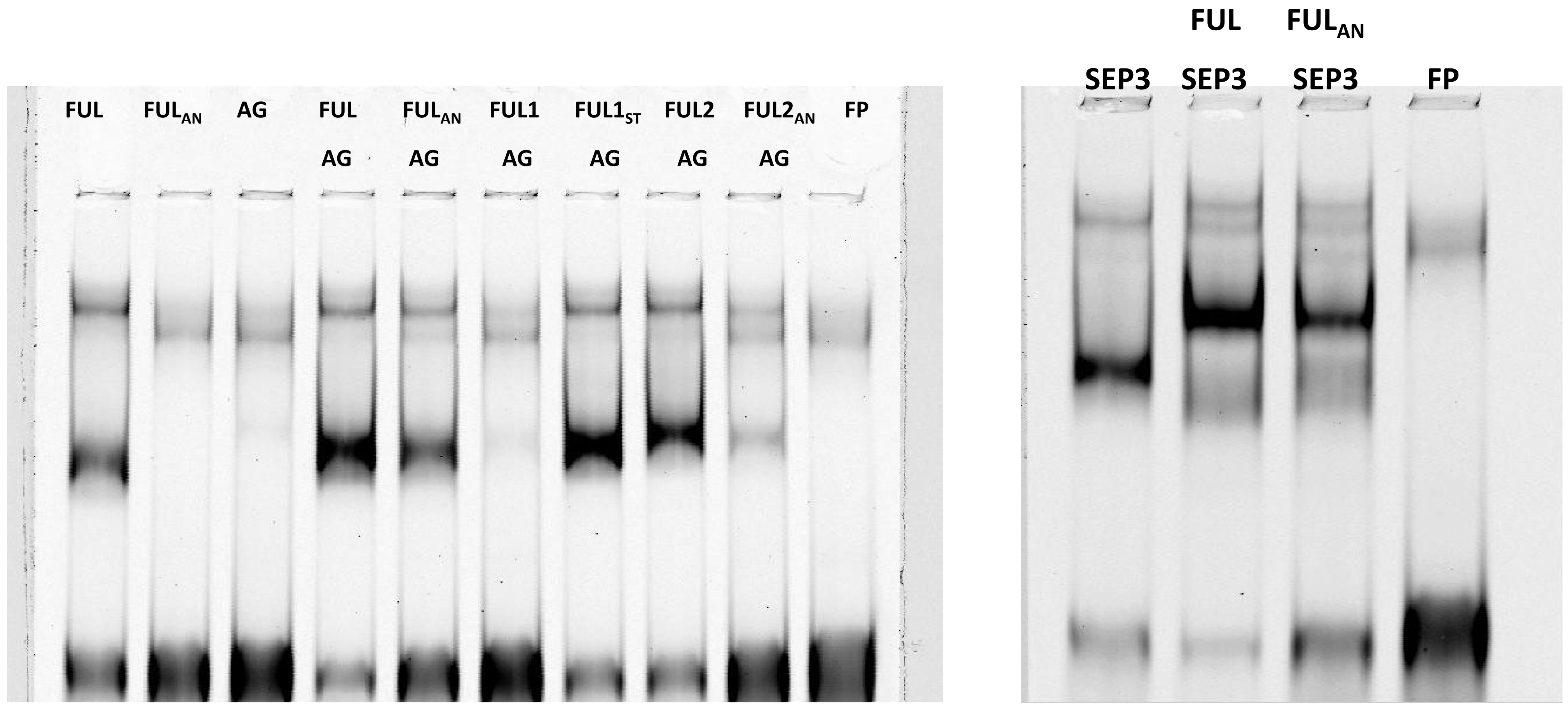

Figure S12. EMSA experiment showing the interaction capacity of the Arabidopsis and tomato FULs and their AN/ST variants, showing that FUL(AN) may bind with higher affinity to AG than FUL2(AN). The canonical *SAUR10* probe was used (Bemer et al., 2017).

Motif	Logo	E-value	# sequences
I region 1	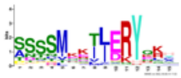	$3.8x10^{-4911}$	11751
I region 2	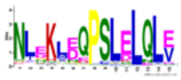	$9.3x10^{-959}$	1616
I region 3	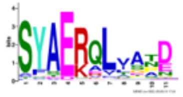	$2.1x10^{-472}$	1276
I region 4	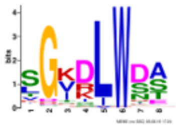	$6.3x10^{-249}$	1112
I region 5	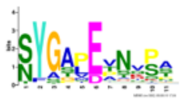	$1.8x10^{-245}$	1134
I region 6	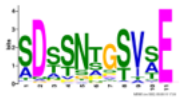	$1.3x10^{-132}$	869
I region 7	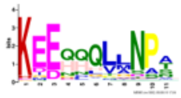	$5.3x10^{-88}$	494
I region 8	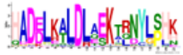	$2.4x10^{-45}$	109
I region 9	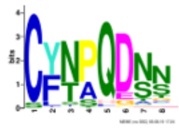	$6.1x10^{-27}$	348
I region 10	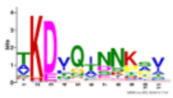	$2.4x10^{-18}$	785
I region 11	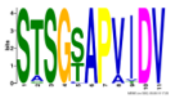	$9.3x10^{-14}$	36
I region 12	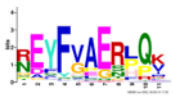	$3x10^{-8}$	93
I region 13	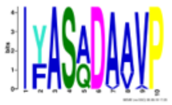	$1.5x10^{-6}$	41
I region 14	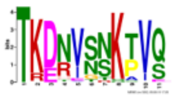	$9.9x10^{-5}$	169
I region 15	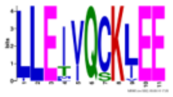	$1.9x10^{-4}$	44
I region 16	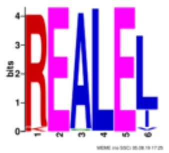	$6.4x10^{-4}$	326
I region 17	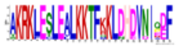	$7.2x10^{-3}$	26
I region 18	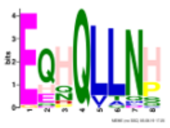	$8x10^{-3}$	398

Figure S13. Table with detected motifs based on the I-regions of 11,966 MADS-domain proteins

Dimer	pTm score	ipTM score	DG^{int}	Buried surface area ($Å^2$)
FUL1-TM3	0.421	0.435	-69	4094
FUL2-TM3	0.403	0.415	-57	3819
FUL2-TAG1	0.404	0.417	-61	3792
FUL-AG	0.411	0.405	-66	3839
FUL-SEP3	0.497	0.493	-66	4137
FUL-SOC1	0.445	0.477	-68	4134
FUL1-TAG1	0.410	0.408	-66	3839
FUL1-FUL1	0.426	0.425	-54	3465
FUL2-FUL2	0.434	0.430	-44	3470
TM3-TM3	0.386	0.389	-53	4092
TAG1-TAG1	0.423	0.420	-67	3803
FUL-FUL	0.420	0.420	-59	3859
SOC1-SOC1	0.463	0.459	-71	3647
AG-AG	0.407	0.401	-64	3817

Figure S14. Table showing the scores of Alphafold2 (AF2) and PISA for the predictions of homo and heterodimers for different combinations of FUL, FUL1, FUL2, AG and SEP3. pTM, predicted template modelling score (AF2), Dg^{int}, free-energy gain during complex formation (PISA). The tomato combinations are in orange boxes.

```
                 10        20        30        40        50        60        70        80
        ....|....|....|....|....|....|....|....|....|....|....|....|....|....|....|....|....|
AGL16   MGRGKIAIKRINNSTSRQVTFSKRRNGLLKKAKELAILCDAEVGVIIFSSTGRLYDFSS~SSMKSVIERYSDAKGETSSENDPAS
SC12    MGRGKIAIKRINNSTSRQVTFSKRRNGLLKKAKELAILCDAEVGVIIFSSTGRLYDFSSNSSMKSVIERYSDAKGETSSENDPAS
ANR1    MGRGKIVIRRIDNSTSRQVTFSKRRSGLLKKAKELSILCDAEVGVIIFSSTGKLYDYASNSSMKTIIERYNRVKEEQHQLLNHAS
SC13    MGRGKIVIRRIDNSTSRQVTFSKRRSGLLKKAKELSILCDAEVGVIIFSSTGKLYDYAS~SSMKTIIERYNRVKEEQHQLLNHAS

                110       120       130       140       150       160       170       180
        ....|....|....|....|....|....|....|....|....|....|....|....|....|....|....|....|....|
AGL16   LHNLQENHRQMMGEELSGLSVEALQNLENQLELSLRGVRMKKDQMLIEEIQVLNREGNLVHQENLDLHKKVNLMHQQNMELHEKV
SC12    LHNLQENHRQMMGEELSGLSVEALQNLENQLELSLRGVRMKKDQMLIEEIQVLNREGNLVHQENLDLHKKVNLMHQQNMELHEKV
ANR1    LQYLQECHRKLVGEELSGMNANDLQNLEDQLVTSLKGVRLKKDQLMTNEIRELNRKGQIIQKENHELQNIVDIMRKENIKLQKKV
SC13    LQHLQECHRKLVGEELSGMNANDLQNLEDQLVTSLKGVRLKKDQLMTNEIRELNRKGQIIQKENHELQNIVDIMRKENIKLQKKV

                210       220       230       240
        ....|....|....|....|....|....|....|....|..
AGL16   TNGLDMRDTSNEHVHLQLSQPQHDHETHSKAIQLNYFSFIA
SC12    TNGLDMRDTSNEHVHLQLSQPQHDHETHSKAIQLNYFSFIA~
ANR1    ISNGTTTYAPPQLQLIQLQPAPREKSIRLGLQLS~~~~~~~~
SC13    ISNGTTTYAPPQLQLIQLQPAPREKSIRLGLQLS~~~~~~~~
```

Figure S15. Alignment showing the paralogs AGL16 and ANR1 and the generated substitution constructs (SCs).

```
                  10        20        30        40        50        60        70        80
          ....|....|....|....|....|....|....|....|....|....|....|....|....|....|....|....|....|
AGL13     MGRGKVEVKRIENKITRQVTFSKRKSGLLKKAYELSVLCDAEVSLIIFSTGGKLYEFSNVGVGRTIERYYRCKDNLLDNDTLEDT
SC14      MGRGKVEVKRIENKITRQVTFSKRKSGLLKKAYELSVLCDAEVSLIIFSTGGKLYEFSNVGVERTIERYYRCKDNLLDNDTLEDT
SC15      MGRGKVEVKRIENKITRQVTFSKRKSGLLKKAYELSVLCDAEVSLIIFSTGGKLYEFSNVGMERTIERYYRCKDNLLDNDTLEDT
AGL6      MGRGRVEMKRIENKINRQVTFSKRRNGLLKKAYELSVLCDAEVALIIFSSRGKLYEFGSVGIESTIERYNRCYNCSLSNNKPEET
SC16      MGRGRVEMKRIENKINRQVTFSKRRNGLLKKAYELSVLCDAEVALIIFSSRGKLYEFGNVGVGSTIERYNRCYNCSLSNNKPEET

                 110       120       130       140       150       160       170       180
          ....|....|....|....|....|....|....|....|....|....|....|....|....|....|....|....|....|
AGL13     ESLLRTHRNLVGEDLEGMSIKELQTLERQLEGALSATRKQKTQVMMEQMEELRRKERELGDINNKLKLETEDH~~DFKGFQDLLL
SC14      ESLLRTHRNLVGEDLEGMSIKELQTLERQLEGALSATRKQKTQVMMEQMEELRRKERELGDINNKLKLETEDH~~DFKGFQDLLL
SC15      ESLLRTHRNLVGEDLEGMSIKELQTLERQLEGALSATRKQKTQVMMEQMEELRRKERELGDINNKLKLETEDH~~DFKGFQDLLL
AGL6      ESLVRTNRNLLGEDLGEMGVKELQALERQLEAALTATRQRKTQVMMEEMEDLRKKERQLGDINKQLKIKFETEGHAFKTFQDLWA
SC16      ESLVRTNRNLLGGDLGEMGVKELQALERQLEAALTATRQRKTQVMMEEMEDLRKKERQLGDINKQLKTKFETEGHAFKTFQDLWA

                 210       220       230       240       250       260
          ....|....|....|....|....|....|....|....|....|....|....|....|
AGL13     ST~~~HQNYISDCNLGYFLQIGFQQHYE~QGEGSSVTKSNARSDAETNFVQ~~~~~~~~~
SC14      ST~~~HQNYISDCNLGYFLQIGFQQHYE~QGEGSSVTWQFPGIAPSFLVQSGLMAAK~~~
SC15      ST~~~HQNYISDCNLGYFLQIGFQQHYE~QGEGSSVTWQFPGIAPSFLVQSGLMAAK~~~
AGL6      ~VEPSHPNV~LDCNTEPFLQIGFQQHYYVQGEGSSVSKSNVAGETNFVQGWVL~~~~~~~
SC16      PVEPSHPNV~LDCNTEPFLQIGFQQHYYVQGEGSSVSKSNVAGETNFVQGWVL~~~~~~~
```

Figure S16. Alignment showing the paralogs AGL6 and AGL13 and the generated substitution constructs (SCs).

```
                10        20        30        40        50        60        70        80
       ....|....|....|....|....|....|....|....|....|....|....|....|....|....|....|....|....|...
SEP3   MGRGRVELKRIENKINRQVTFAKRRNGLLKKAYELSVLCDAEVALIIFSNRGKLYEFCSS-SSMLRTLERYQKCNYGAPEPNVPSREA
SEP4   MGRGKVELKRIENKINRQVTFAKRRNGLLKKAYELSVLCDAEIALLIFSNRGKLYEFCSSPSGMARTVDKYRKHSYATMDPNQSAKDL
SC10   -GRGKVELKRIENKINRQVTFAKRRNGLLKKAYELSVLCDAEIALLIFSNRGKLYEFCSS-SGMARTVDKYRKHSYATMDPNQSAKDL
SC11   -GRGKVELKRIENKINRQVTFAKRRNGLLKKAYELSVLCDAEIALLIFSNRGKLYEFCSS-SSMARTVDKYRKHSYATMDPNQSAKDL

               130       140       150       160       170       180       190       200
       ....|....|....|....|....|....|....|....|....|....|....|....|....|....|....|....|....|...
SEP3   LGPLSTKELESLERQLDSSLKQIRALRTQFMLDQLNDLQSKERMLTETNKTLRLRLAD----------GYQMPLQLNPNQEEVDHYGR
SEP4   LSEMDVNELEHLERQVDASLRQIRSTKARSMLDQLSDLKTKEEMLLETNRDLRRKLEDSDAALTQSFWGSSAAEQQQQHQQQQQGMSS
SC10   LSEMDVNELEHLERQVDASLRQIRSTKARSMLDQLSDLKTKEEMLLETNRDLRRKLEDSDAALTQSFWGSSAAEQQQQHQQQQQGMSS
SC11   LSEMDVNELEHLERQVDASLRQIRSTKARSMLDQLSDLKTKEEMLLETNRDLRRKLEDSDAALTQSFWGSSAAEQQQQHQQQQQGMSS

               250       260
       ....|....|....|....|....|...
SEP3   MGA------GPSVNNYMLGWLPYDTNSI
SEP4   ANATNSATTSQNVNGFFPGWMV------
SC10   ANATNSATTSQNVNGFFPGWMV------
SC11   ANATNSATTSQNVNGFFPGWMV------
```

Figure S17. Alignment showing the close homologs SEP3 and SEP4 and the generated substitution constructs (SCs).

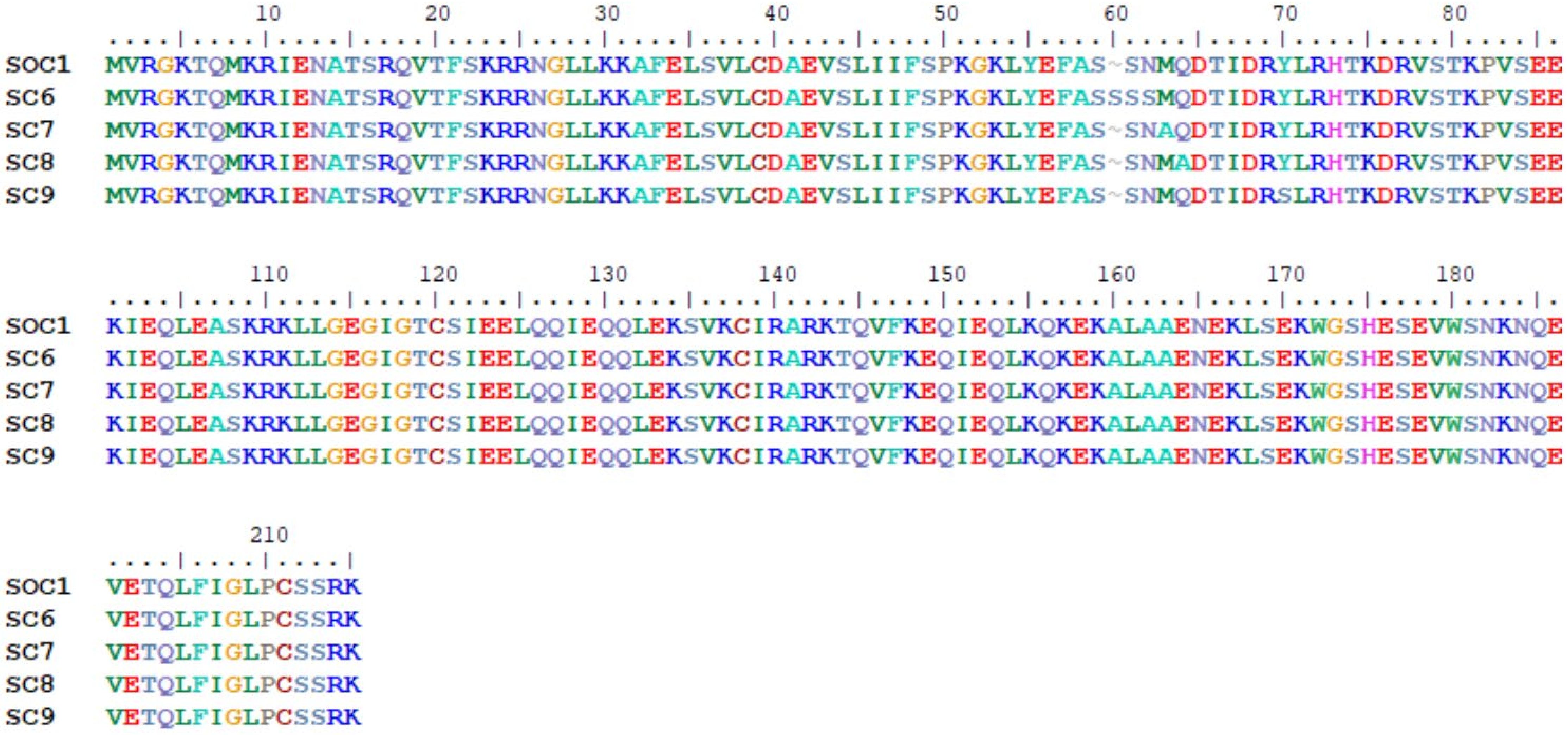

Figure S18. Alignment of SOC1 with the generated substitution constructs to test the Met/Tyr/loop hypothesis.

```
                10        20        30        40        50        60        70        80        90
       ....|....|....|....|....|....|....|....|....|....|....|....|....|....|....|....|....|....|
SEP3   MGRGRVELKRIENKINRQVTFAKRRNGLLKKAYELSVLCDAEVALIIFSNRGKLYEFCSSSSMLRTLERYQKCNYGAPEPNVPSREALAV
SC2    MGRGRVELKRIENKINRQVTFAKRRNGLLKKAYELSVLCDAEVALIIFSNRGKLYEFC-SSSMLRTLERYQKCNYGAPEPNVPSREALAV
SC1    MGRGRVELKRIENKINRQVTFAKRRNGLLKKAYELSVLCDAEVALIIFSNRGKLYEFC-SSNMLRTLERYQKCNYGAPEPNVPSREALAV

               100       110       120       130       140       150       160       170       180
       ....|....|....|....|....|....|....|....|....|....|....|....|....|....|....|....|....|....|
SEP3   ELSSQQEYLKLKERYDALQRTQRNLLGEDLGPLSTKELESLERQLDSSLKQIRALRTQFMLDQLNDLQSKERMLTETNKTLRLRLADGYQ
SC2    ELSSQQEYLKLKERYDALQRTQRNLLGEDLGPLSTKELESLERQLDSSLKQIRALRTQFMLDQLNDLQSKERMLTETNKTLRLRLADGYQ
SC1    ELSSQQEYLKLKERYDALQRTQRNLLGEDLGPLSTKELESLERQLDSSLKQIRALRTQFMLDQLNDLQSKERMLTETNKTLRLRLADGYQ

               190       200       210       220       230       240       250
       ....|....|....|....|....|....|....|....|....|....|....|....|....|....|.
SEP3   MPLQLNPNQEEVDHYGRHHHQQQQHSQAFFQPLECEPILQIGYQGQQDGMGAGPSVNNYMLGWLPYDTNSI
SC2    MPLQLNPNQEEVDHYGRHHHQQQQHSQAFFQPLECEPILQIGYQGQQDGMGAGPSVNNYMLGWLPYDTNSI
SC1    MPLQLNPNQEEVDHYGRHHHQQQQHSQAFFQPLECEPILQIGYQGQQDGMGAGPSVNNYMLGWLPYDTNSI
```

Figure S19. Alignment of SEP3 with the generated substitution constructs (SC) to test the Met/Tyr/loop hypothesis.

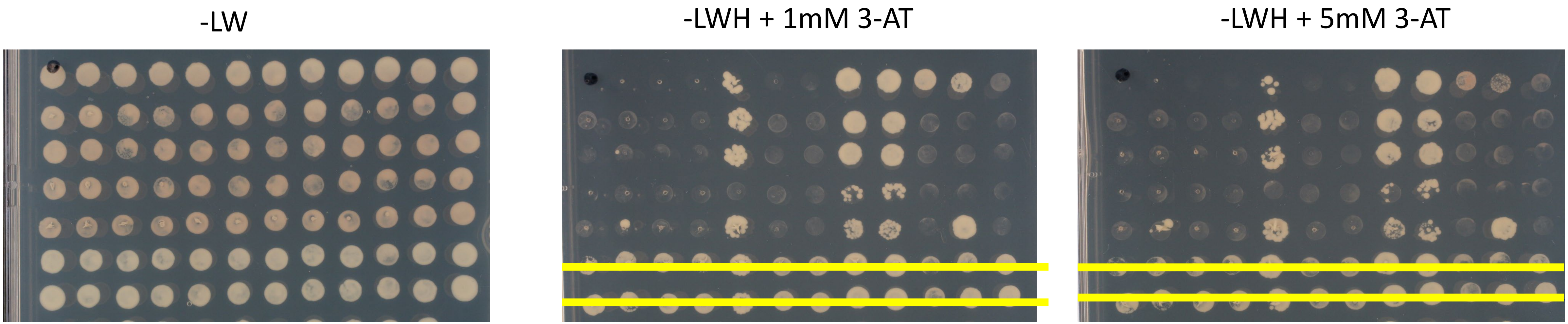

	pDEST22	ANR1	AGL42	SVP	AGL21	SOC1	AGL17	AGL25/FLC	AGL16	AGL15	AGL14	AGL13	CAL
	pDEST32	1	2	3	4	5	6	7	8	9	10	11	12
A1	**SOC1**												
B1	**SC6**												
C1	**SC7**												
D1	**SC9**												
E1	**SEP3**												
F1	**SC1**												
G1	**SC2**												

Not reliable due to autoactivation (SC1 and SC2 based on full-length SEP3)

Figure S20. Yeast two-hybrid results scored after 7 days at 20 °C. The table indicates the positions of the different clones. The top row indicates the pDEST22 (prey) clones, the column the pDEST32 (bait) clones. Yellow lines indicate spots that were not scored due to either autoactivation or bad growth on the control (-LW) plate. Scoring was based on the –LWH +5mM 3-AT plate.

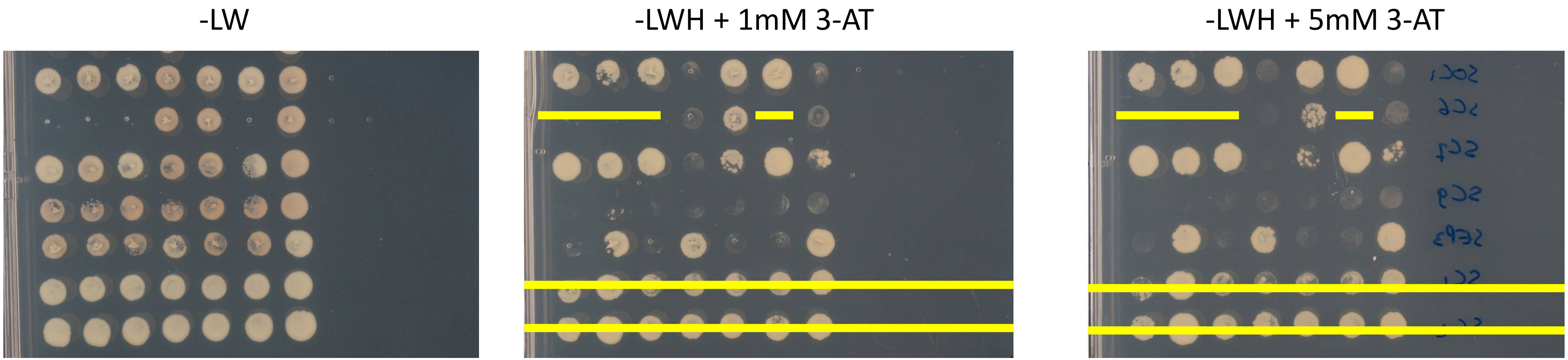

	pDEST22	dSEP3	FUL	AP1	SHP2	SEP2	SHP1	AG					
	pDEST32	1	2	3	4	5	6	7	8	9	10	11	12
A1	SOC1												
B1	SC6												
C1	SC7												
D1	SC9												
E1	SEP3								Less growth on control plate				
F1	SC1												
G1	SC2												

Not reliable due to absence of growth on control plate or autoactivation

Figure S21. Yeast two-hybrid results scored after 7 days at 20 °C. The table indicates the positions of the different clones. The top row indicates the pDEST22 (prey) clones, the column the pDEST32 (bait) clones. Yellow lines indicate spots that were not scored due to either autoactivation or bad growth on the control (-LW) plate. Scoring was based on the –LWH +5mM 3-AT plate.

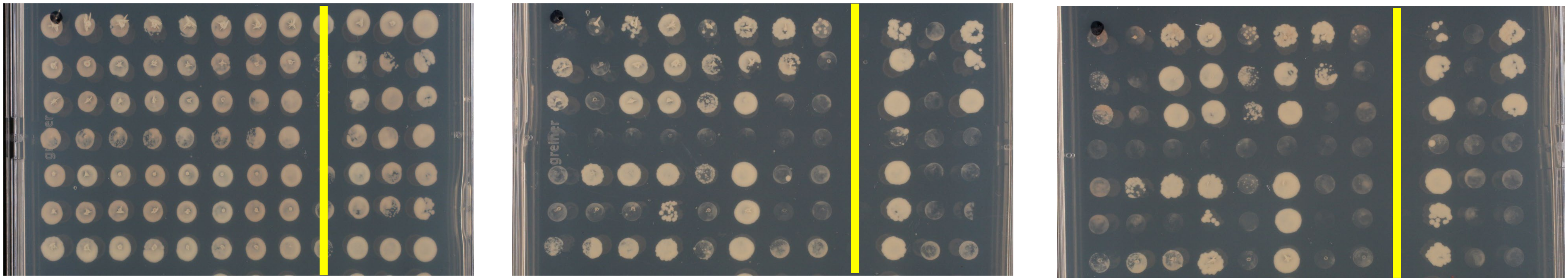

	pDEST32	ANR1	AGL42	AGL24	SVP	AGL21	SOC1	AGL19	AGL17	AGL25/FLC	AGL16	AGL15	AGL14
	pDEST22	1	2	3	4	5	6	7	8	9	10	11	12
A1	SOC1												
B1	SC6												
C1	SC7												
D1	SC9												
E1	SEP3												
F1	SC1												
G1	SC2												

Not reliable due to absence of growth on control plate or autoactivation

Figure S22. Yeast two-hybrid results scored after 7 days at 20 °C. The table indicates the positions of the different clones. The top row indicates the pDEST32 (bait) clones, the column the pDEST22 (prey) clones. Yellow lines indicate spots that were not scored due to either autoactivation or bad growth on the control (-LW) plate. Scoring was based on the –LWH +5mM 3-AT plate.

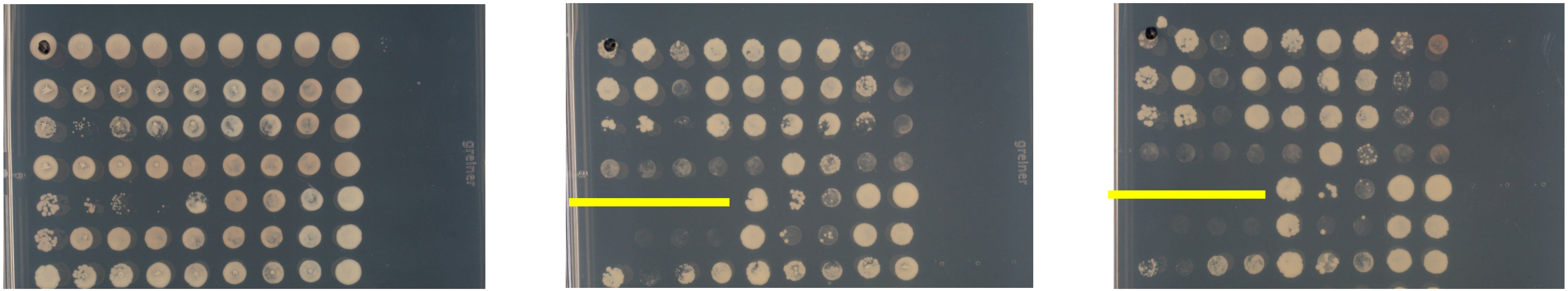

**pDEST32**	AGL13	AGL11/STK	CAL	dSEP3	FUL	SEP2	SEP4	SHP1	AG			
pDEST22	1	2	3	4	5	6	7	8	9	10	11	12
SOC1												
SC6												
SC7												
SC9												
SEP3												
SC1												
SC2												

Not reliable due to absence of growth on control plate or autoactivation

Figure S23. Yeast two-hybrid results scored after 7 days at 20 °C. The table indicates the positions of the different clones. The top row indicates the pDEST32 (bait) clones, the column the pDEST22 (prey) clones. Yellow lines indicate spots that were not scored due to either autoactivation or bad growth on the control (-LW) plate. Scoring was based on the –LWH +5mM 3-AT plate.
